# Comparative ^1^H NMR-Based Chemometric Evaluations of the Time-Dependent Generation of Aldehydic Lipid Oxidation Products in Culinary Oils Exposed to Laboratory-Simulated Shallow Frying Episodes: Differential Patterns Observed for Omega-3 Fatty Acid-Containing Soybean Oils

**DOI:** 10.3390/foods10102481

**Published:** 2021-10-17

**Authors:** Angela I. Wann, Benita C. Percival, Katy Woodason, Miles Gibson, Siâny Vincent, Martin Grootveld

**Affiliations:** 1 Leicester School of Pharmacy, De Montfort University, The Gateway, Leicester LE1 9BH, UK; Angela.Allen@dmu.ac.uk (A.I.W.); benita.c.percival@dmu.ac.uk (B.C.P.); katy.woodason@dmu.ac.uk (K.W.); miles.gibson@dmu.ac.uk (M.G.); P2449250@my365.dmu.ac.uk (S.V.); 2 School of Life Sciences, Pharmacy and Chemistry, Kingston University, River House, 53–57 High Street, Kingston upon Thames KT1 1LQ, UK

**Keywords:** soybean oil, frying oils, high-temperature frying practices, lipid oxidation products, aldehydes, food toxicology, ^1^H NMR analysis, ω-3 fatty acids, low-molecular-mass *n*-alkanals, 4-Oxo-*n*-alkanals, chemometrics analysis

## Abstract

Soybean oil is the second most exported oil from the United States and South America, and is widely marketed as a cooking oil product containing numerous health benefits for human consumers. However, culinary oils with high polyunsaturated fatty acid (PUFA) contents, are known to produce high quantities of lipid oxidation products (LOPs), including toxic aldehydes upon exposure to high-temperature frying episodes. Previous studies have demonstrated causal links between aldehyde ingestion and inhalation with deleterious health perturbations, including mutagenic and carcinogenic effects, along with cardiovascular and teratogenic actions. In this study, aldehydic LOPs were detected and quantified in commercially available samples of soybean, avocado, corn and extra-virgin olive oil products before and after their exposure to laboratory-simulated laboratory frying episodes (LSSFEs) using high-resolution 1H nuclear magnetic resonance (NMR) analysis. Results acquired demonstrated that PUFA-rich soybean and corn oils gave rise to the highest concentrations of oil aldehydes from the thermo-oxidation of unsaturated fatty acids, whereas monounsaturated fatty acid (MUFA)-laden avocado and olive oils were much more resistant to this peroxidation process, as expected. Multivariate chemometrics analyses provided evidence that an orthogonal component pattern of aldehydic LOPs featuring low-molecular-mass n-alkanals such as propanal, and 4-oxo-alkanals, arises from thermo-oxidation of the ω-3 fatty acid (FA) linolenic acid (present in soybean oils at levels of ca. 7% (*w*/*w*)), was able to at least partially distinguish this oil from corresponding samples of thermally-stressed corn oil. Despite having a similar total PUFA level, corn oil has only a negligible ω-3 FA content, and therefore generated significantly lower levels of these two aldehyde classes. In view of the adverse health effects associated with dietary LOP ingestion, alternative methodologies for the incorporation of soybean oils within high-temperature frying practices are proposed.

## 1. Introduction

Culinary frying oils which contain high contents of unsaturated fatty acids (UFAs), particularly polyunsaturated fatty acids (PUFAs), unfortunately oxidatively deteriorate when exposed to high-temperature frying practices at ca. 180 °C, most especially when reused for this purpose [1]. Indeed, this degradation arises from the ready susceptibility of UFAs therein to the autocatalytic, self-perpetuating lipid peroxidation process during such frying episodes. Moreover, this mechanistically complex reaction process also occurs during prolonged periods of culinary oil storage and exposure to light, notably at temperatures higher than room temperature, as is often the case in third-world countries with tropical or sub-tropical climates [1,2]. Additionally, the type of frying practice employed can directly impact on the peroxidation of UFAs present in culinary oils [3,4,5]. This reaction involves highly reactive free radical species and/or a singlet oxygen (^1^O_2_) interacting with the *bis*-allylic- or allylic-CH_2_- groups located between or adjacent to carbon-carbon double bond locants of long PUFA, or monounsaturated fatty acid (MUFA) chains, respectively, which consecutively gives rise to primary and then secondary lipid oxidation products (LOPs). The mechanisms of formation of such LOPs, and the magnitudes of concentrations formed are critically dependent on the precise molecular nature of UFAs and hence the extent of thermally induced peroxidation [6]. The production of primary LOPs, particularly lipid hydroperoxides, including hydroperoxymonoenes (HPMs) and conjugated hydroperoxydienes (CHPDs), is therefore critically dependent on the fatty acid (FA) contents of culinary oils employed for frying practices, the former being derived from oleoylglycerols, and the latter from linoleoyl- and more highly unsaturated linolenoylglycerols [1,3,6]. Since these primary products are chemically unstable at high frying temperatures, they fragment to a wide range of secondary LOPs, including alcohols, ketones, oxoacids, alkenes and alkanes; however, aldehydes, both saturated and unsaturated, represent the most important of these secondary LOPs in view of their high levels of generation and adverse toxicological properties potentially exertable in humans consuming fried foods, or those inhaling such toxins during frying practices [1,5]. Further LOPs include a series of toxic epoxy-fatty acids [1,2]. 

Culinary frying oils with high PUFA contents are the most susceptible to thermo-oxidation, whereas those with high saturated fatty acid (SFA) levels are the least so [1,7]; indeed, SFAs are virtually completely resistant to peroxidation. Although also prone to peroxidation, MUFAs are much more resistant to this process than PUFAs, and therefore culinary oils which are rich in MUFAs such as olive or avocado oils have been found to generate lower or much lower concentrations of aldehydic LOPs than PUFA-laden ones. These LOPs are not only harmful to health, but can also affect the taste, appearance and viability for use of commercially available culinary frying oils. Hence, lipid peroxidation represents a major cause for concern to manufacturers, restaurateurs and human consumers [1,2]. 

Soybean oils are often consumed as food products in margarines, shortenings and salad dressings, and are frequently used for frying/cooking purposes, a development which is at least partially attributable to its high flash-point (315 °C). Moreover, this cooking oil has been marketed with numerous health benefits since it is high in ω-6 (linoleoylglycerol), and ω-3 (linolenoylglycerol) contents (*ca.* 57 and 7% (*w*/*w*), respectively). However, in view of its significant ω-3 FA content, and the knowledge that this class of PUFAs peroxidises at a faster rate than ω-6 FAs [6] (almost exclusively present as linoleoylglycerols in vegetable oils), we may expect a more rapid rate of peroxidation and perhaps higher levels of LOPs generated in soybean oil over those derived from oils with similar total PUFA levels (e.g., corn oil) when exposed to high-temperature frying practices conducted under identical conditions. Moreover, the homologue status and patterns of different classes of LOPs generated, notably aldehydes, are expected to be distinct from those arising from the peroxidation of ω-6 FAs [8,9]. 

Studies exploring the peroxidation of soybean oil have been performed by a range of research groups throughout the last 25 years [10,11], including one major study by our group published in 1995 [3]. However, one such early investigation was limited to the assessment of soybean oil peroxidation during storage conditions (65 °C for a 30-day duration), using ^1^H nuclear magnetic resonance (NMR) and simple spectrophotometric analyses [10]. Additionally, the peroxidation markers peroxide value, along with conjugated diene, triene and *p*-anisidine indices were also spectrophotometrically determined in that study, as were thiobarbituric acid-reactive substances (TBARS). Although ^1^H NMR analysis was indeed utilised by these researchers, it was only conducted at an operating frequency of 300 MHz, and was used only to monitor the oxidative degradation of oil PUFAs and not LOP production during the above periods of storage [10]. 

Our 1995 study [3] first monitored the primary and secondary oxidation products in soybean and other culinary oils, during laboratory-simulated frying episodes of 30, 60 and 90 min using both 400 and 600 MHz ^1^H NMR spectrometers. Indeed, we were the first to identify *n*-alkanals, (*E*)-2-alkenals and (*E*,*E*)-alka-2,4-dienals and their isomeric conjugated hydroperoxydiene precursors in such frying oil products using this technique. Once high-field ^1^H NMR analysis was established as a successful method for monitoring aldehydes [3,11], Gullien and Ruiz (2003) [12] expanded upon its application and used it to distinguish between the nature and concentrations of these secondary LOPs in different edible oils during their exposure to heating episodes, these studies also featuring soybean oil. Moreover, further experiments performed by this group considered the generation of LOPs in this and other cooking oils during deep-frying heating episodes at 190 °C using ^1^H NMR spectroscopic analysis [13]. Results acquired showed higher concentrations of oxygenated α,β-unsaturated aldehydes formed in soybean oil when compared to those formed in other oils of lower PUFA contents such as olive oil, although this is exactly what would be expected. However, such classes of unsaturated aldehydes are also produced from the peroxidation of linoleoyl- as well as linolenoylglycerols, and although high-resolution ^1^H NMR analysis can readily distinguish between these, it is less able to differentiate between individual homologues within the same classes of these LOPs. However, at operating frequencies of 500 MHz or above, which have a much improved spectral resolution than that achievable at only 400 MHz, it does have the ability to distinguish between the lower and higher homologues of *n*-alkanal LOPs, which have well-resolved, separate -CHO function triplet resonances, the former located downfield of the latter by ca. 0.10 ppm, and therefore this information was exploited in order to determine low-molecular-mass (LMM) *n*-alkanals, which are secondary LOPs specifically derived from the fragmentation of hydroperoxides arising from the thermo-oxidation of ω-3 FAs. For this purpose, we used an operating frequency of 600 MHz, which also enhances the sensitivity of the ^1^H NMR technique, and therefore relatively low levels of LOPs generated from the peroxidation of UFAs with only low oil contents, including linolenic acid in soybean oil, are much more readily detectable and quantifiable than they are when using a 400 MHz spectrometer. 

To date, it appears that all NMR-based studies focused on the thermo-oxidation of soybean oil have not reported any differences between the patterns of different classes of aldehydic LOPs generated therein from those of other frying oils, notably those with similar PUFA contents but which are virtually devoid of linolenic acid, such as corn oil. Similarly, MUFA-rich avocado and olive oils all contain only 0.80–1.20% (*w*/*w*) of this FA, and this justified their selection for the current study. 

A further, very recent investigation focused on the peroxidative stability of soybean oil when exposed to accelerated storage conditions at 70 °C reported by Martin-Rubio et al. [14], however, also did not involve the analysis of any ω-3 FA-specific LOPs. Indeed, the ^1^H NMR data available in this investigation was limited to aldehydes which arise from the fragmentation of both ω-3 and much more predominant ω-6 PUFA hydroperoxides, together with those from MUFA-derived HPMs. However, the ^1^H NMR analysis conducted in this study was performed at an operating frequency of only 400 MHz, which, as noted above, unfortunately limits the detection of some aldehydic LOPs, particularly those present at low or very low concentrations, including LMM *n*-alkanals such as *n*-propanal, along with acrolein, which are derived from ω-3 FA peroxidation. 

Therefore, in the current study, two soybean oil, in addition to two extra-virgin olive oil, one corn oil and one avocado oil products, which are all commercially available, were analysed using ^1^H NMR spectroscopic analysis in order to investigate the formation of toxic secondary aldehydic LOP species both before and during high-temperature laboratory-simulated shallow frying episodes (LSSFEs). Both univariate (UV) and multivariate (MV) NMR-based chemometric strategies were employed to explore distinctions between the oils evaluated. Corresponding thermally-induced changes to the lipid profiles and FA compositions of these culinary oils during LSSFEs were also investigated. 

Major Study Objectives:

(1) To examine the susceptibility of two soybean oil products to peroxidation induced by their exposure to LSSFEs for periods of up to 90 min., and to comparatively assess the nature and levels of aldehydic LOPs generated therein with those produced from the thermal stressing of other culinary oils, specifically corn, avocado and extra-virgin olive oils;
(2) To conduct both UV and MV forms of chemometrics analysis to determine the pattern of aldehydic LOPs generated from thermo-oxidised soybean oil products, with a view to exploring those specifically arising from the peroxidation of ω-3 FAs substrates therein, and to distinguish these from those produced in other culinary oils, notably corn oil which has a similar total PUFA, but negligible ω-3 FA content;
(3) To consider and discuss technologies available for rendering soybean oil as a safer, virtually health risk-free frying medium.

## 2. Materials and Methods

### 2.1. Materials and Culinary Oil Products Investigated

Deuterochloroform (C^2^HCl_3_) containing 3% (*w*/*w*) tetramethylsilane (TMS), 1,3,5-trichlorobenzene (TCB), authentic reference aldehydes and 2,5-di-*tert-*butylhydroquinone (2,5-DTBHQ) were all purchased from Sigma-Aldrich Chemical Co. Ltd. (Gillingham, UK). Culinary oils were purchased from commercial retail outlets in the USA. Samples were retained cold and unexposed to light during transport to our laboratory for ^1^H NMR analysis. Samples were stored under dark conditions in the laboratory at ambient temperature to reduce photodegradation and oxidation prior to ^1^H NMR and further analyses conducted in our laboratory. Label-specified % (*w*/*w*) SFA, MUFA and PUFA contents of these oils were: Spanish extra-virgin olive oil (SEVOO1), 16.3, 75.7 and 8.0%; Italian extra-virgin olive oil (IEVOO1), 17.0, 76.0 and 7.0%; Avocado oil (AVO1), 16.0, 70.5 and 13.5%; Corn oil (CO1), 12.9, 27.6 and 59.5%; Soybean oil 1 (SBO1) 16.8, 26.9 and 56.3%; Soybean oil 2 (SBO2) 17.7, 24.1 and 58.2%, respectively. Of the 2 soybean oil products, the first (SBO1) was described as an organic expeller-pressed vegetable oil, the second (SBO2) as a refined soybean vegetable oil (Supplementary Materials Section Table S1). Expeller pressing represents a mechanical process for the extraction of culinary oils from raw materials; friction generated during the pressing of the raw soybean material yields heat. 

### 2.2. Exposure of Culinary Oils to LSSFEs 

For each of the above culinary oils evaluated, 6.00 mL volumes of each product were placed in clean air-dried 250 mL glass beakers within a thermostatted silicon oil bath which was heated to 180 °C in the presence of atmospheric O_2_. Aliquots (~0.30 mL) of oil were sampled at 0, 5, 10, 20, 30, 60 and 90 min. thermal stressing time intervals as performed in [1], and each experiment was repeated in triplicate for all oils investigated. 

### 2.3. ^1^H NMR Analysis: Sample Preparation, and Spectral Acquisition Parameters

A 200 µL aliquot of each oil sample collected was then transferred to a 1.50 mL microcentrifuge tube, to which was then immediately added 100 µL of a C^2^HCl_3_ solution containing 2,5-DTBHQ (5.00 mmol./L), a chain-breaking antioxidant, in order to prevent any further, artefactual peroxidation of oil PUFAs during laboratory preparation and spectral acquisition episodes. These mixtures were then further diluted through the addition of 400 µL of TMS-containing C^2^HCl_3_, the latter of which was also employed as a deuterated field frequency lock solvent; TMS served as a chemical shift reference (δ = 0.00 ppm). Moreover, a 60 µL volume of TCB solution (66.0 mmol./L), was utilised as an internal concentration standard (*s*, δ = 7.26 ppm). Resulting analysis solutions were then thoroughly rotamixed and then transferred to 5-mm diameter NMR tubes. 

^1^H NMR spectra were acquired on a 600 MHz Bruker AV NMR spectrometer operating at a frequency of 600.13 MHz (School of Life Sciences, Kingston University, Kingston, London, UK). Each sample was analysed using the zg30 pulse program, a 30° flip angle, with 128 scans and 2 dummy scans; a total of 65,536 data points were acquired, using an acquisition time of 2.65 s. Samples were inserted onto a 60-sample autosampler for ^1^H NMR analysis. Resonances were assigned by a consideration of chemical shifts, coupling patterns and coupling constants, and comparisons with those of reference spectra available in the scientific literature, e.g., [1,2,15,16,17]. The acquisition of ^1^H-^1^H correlation (COSY) and total correlation (TOCSY) spectra served to confirm these assignments.

### 2.4. Experimental Design and Statistical Analysis of Time-Dependent Aldehyde Production in Different Culinary Oils Exposed to LSSFEs: Univariate and MV Chemometrics Analysis 

Univariate statistical analysis conducted to detect significant differences between the mean replicate values of the concentrations of all nine identified aldehydes for each oil product and at each LSSFE sampling time-point was performed by analysis of variance (ANOVA). The experimental design employed comprised a two-factor model with the fixed effects of culinary oil product (O*_i_*) and LSSFE sampling time-point (T*_j_*). Also incorporated in the design was a first-order oil product x sampling time-point interaction effect (OT*_ij_*). The mathematical model for this design is provided in Equation (1), where y*_ijk_* represents each replicate aldehyde concentration, μ the overall sample mean aldehyde concentration in the absence of any contributory sources of variation, and e*_ijk_* fundamental error. Software employed for this analysis was *XLSTAT2014* or *2020* (Addinsoft, Paris, France). *Post-hoc* evaluations of oil types involved comparisons of their least square mean (LSM) values using the Bonferroni test.

y_ijk_ = μ + T*_j_* + O*_i_* + OT*_ij_* + e*_ijk_* (1)

An equivalent experimental design and statistical analysis approach was also employed for exploring variance contributions from the T*_j_*, O*_i_*, and OT*_ij_* factors towards the FA compositions of each culinary oil investigated, i.e., their total SFA, MUFA, PUFA, UFA and ω-3 FA contents.

Comparisons of the ratios of low- to high-molecular-mass *n*-alkanal concentrations between the two soybean oil and the single corn oil products at the 90 min LSSFE time-points were performed by ANOVA, which was coupled with the robust Welch test statistic to circumvent any problems potentially arising from the heterogeneity of intra-sample variances. 

Principal component analysis (PCA) was performed on the full ^1^H NMR-based aldehyde concentration dataset featuring all aldehyde concentrations at all LSSFE time-points (0–90 min) for all 6 oils investigated, or alternatively this MV analysis approach was restricted to the 90 min sampling time-point only. However, partial least-squares discriminant analysis (PLS-DA), and orthogonal projections to latent structures discriminant analysis (OPLS-DA) were performed on the 90 min LSSFE time-point only aldehyde level dataset. Replicate analyses (*n* = 3) of each aldehyde per oil product were evaluated. These MV analyses were conducted using either *XLSTAT2014* or *2020*, or *Metaboanalyst 5.0* (University of Alberta and National Research Council, National Institute for Nanotechnology (NINT), Edmonton, AB, Canada) software module options. Datasets (mmol. aldehyde/mol. FA) were analysed following generalised logarithmic (glog)-transformation and Pareto scaling. Heatmaps coupled with agglomerative hierarchical output variable (aldehyde structural nature) clusterings, and correlation feature diagrams were also generated with *Metaboanalyst 5.0* software. 

## 3. Results and Discussion

### 3.1. ^1^H NMR Analysis of the FA Contents of Culinary Oils before and after Exposure to LSSFEs

**Figure 1:**
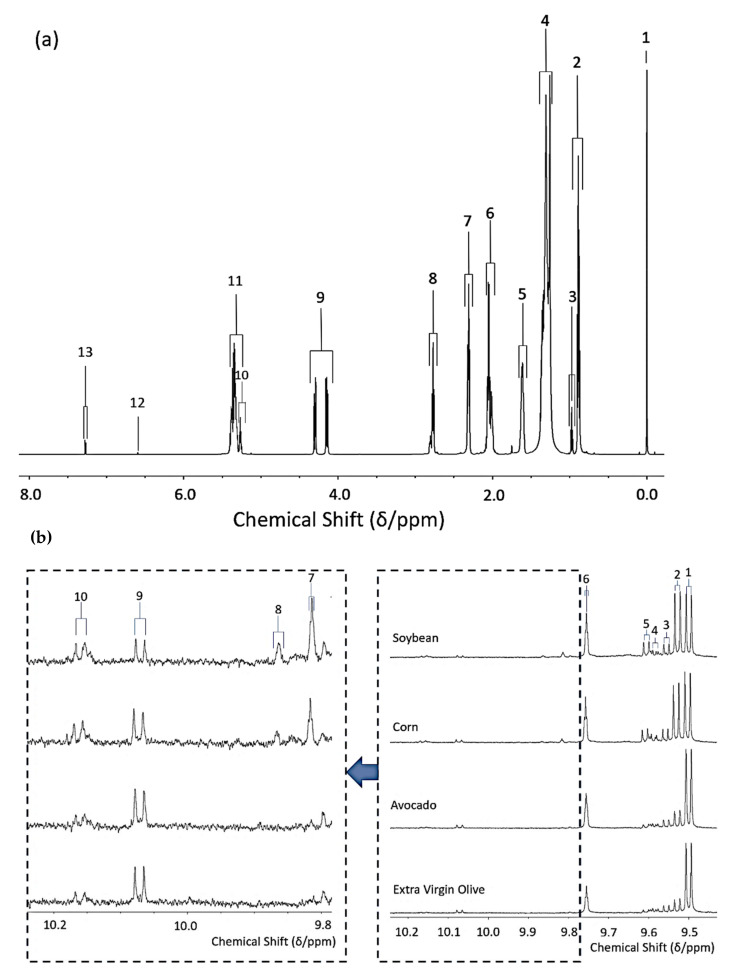
(**a**) Typical complete 600 MHz ^1^H NMR spectrum acquired on soybean oil prior to exposure to LSSFEs, showing the major lipidic acyl chain group resonances; associated chemical shift values, multiplicities and assignments of resonances therein are detailed in Table 1. (**b**) 600 MHz ^1^H NMR spectra highlighting the expanded aldehydic region (9.40–10.30 ppm) with typical spectra acquired on soybean, corn, avocado and olive oil samples after exposure to a LSSFE for a 90 min period at 180 °C; also shown are the expanded 9.76–10.30 ppm regions of these spectra (numerical labels correspond to the assignments available in Table 3, as reported in [1]).

Figure 1a shows the major triacylglycerol (TAG) resonances detectable in the ^1^H NMR profile of a typical soybean oil sample as an example. Full assignments for each of these signals are provided in Table 1, and these were based on their chemical shift values, coupling patterns and coupling constants as previously reported [1,3,15,16,17]. 

In order to determine the lipid compositional profiles of the cooking oils evaluated, their MUFA, PUFA and SFA (molar %) contents were computed and monitored at increasing time-points throughout the complete 90 min thermal stressing cycle. Major TAG lipid signals were normalized to that of the total FA terminal-CH_3_ groups (*t*, δ = 0.84–0.99 ppm). Signals employed for these calculations were the unsaturated ω-3 FA acyl group-terminal-CH_3_ (*t*. δ = 0.95–0.99 ppm); FA acyl group -CH=CH-CH_2_- (*m*, δ = 1.96–2.10 ppm); *bis*-allylic-CH=CH-CH_2_-CH=CH- protons of linoleoylglycerols (*m*, δ = 2.75–2.79 ppm); ω-3 FA acyl group *bis*-allylic-CH=CH-CH_2_-CH=CH- protons of linolenoylglycerols (*m*, δ = 2.79–2.82 ppm), and the -CH_2_OH functions of TAG backbones (*dd,dd*, δ = 4.12–4.32 ppm) as documented in [17] (Table 1). Moreover, Table S1 in the Supplementary Materials section provides a full listing of the ^1^H NMR-determined FA compositions at the 0, 5, 10, 20, 30, 60 and 90 min LSSFE time-points for all six oils monitored. In view of the very low contents of ω-3 FAs present in extra-virgin olive, avocado and corn oils (i.e., <1.2 weight % for each of them), it was not possible to monitor LSSFE-induced changes in its contents in these products in view of the superimposition of its characteristic terminal-CH_3_ group resonance at δ = 0.95–0.99 ppm on that of the similarly intense ^13^C satellite of the ω-6 FA signal (δ = 0.84–0.91 ppm). Therefore, only the weight % literature values for the unheated oils are provided in Table S1 for comparative purposes.

An evaluation of the oil FA compositions prior to and following thermal stressing episodes (Table S1, Supplementary Materials) demonstrated that the greatest thermally induced modifications to their lipid composition profiles were observed for both soybean oils tested, and corn oil, in view of their relatively high PUFA contents. Notably, the relative SFA content levels of the oils increased significantly in these products in view of a corresponding decrease in overall PUFA and, to a much lesser extent, MUFA contents. However, as expected, time-dependent decreases in overall UFA contents were evident across all six oils tested, although markedly greater decreases in total PUFA contents were observed in corn and soybean oils, as expected. Indeed, their total PUFA levels were reduced by 5–10 molar % at the 90 min LSSFE time-point. Culinary oils with higher MUFA contents, however, exhibited the smallest FA compositional changes post-thermal stressing, i.e., those observed for the two extra-virgin olive oils investigated. Avocado oil, however, underwent some moderate changes in its composition, specifically increases in its SFA (>7%) and decreases in its PUFA (>3%) molar contents, although such changes were not as prominent as those observed for soybean and corn oils. These modifications in FA concentrations demonstrate the high susceptibilities of culinary oil PUFAs to thermo-oxidation and degradation during LSSFEs, and also highlight the fact that MUFA-rich oils display a much greater resistance against oxidative deterioration. Hence, from a toxicological standpoint, these high MUFA content oils are generally more suitable for commercial or domestic high-temperature frying practices. For the total UFA and SFA contents, the orders of decrease and increase in their magnitudes were SBO2 ≈ SBO1 ≈ CO1 > IEVOO1 ≈ AVO1 > SEVOO1 (starting from the 10 min time-point), and CO1 ≈ SBO2 > SBO1 > IEVOO1 ≈AVO1 > SEVOO1 (from the 20 min time-point), respectively. 

ANOVA of the FA composition datasets revealed extremely highly significant differences between both oil type and sampling time-point (0–90 min) mean values (Table 2). Indeed, such differences had *p* values ranging from <10^−177^ to <10^−23^ (oil type), and <10^−51^ to <10^−25^ (LSSFE time-point). Bar diagrams of these mean values, with their associated 95% confidence intervals (CIs), are shown in Figure S1 of the Supplementary Materials section. Interestingly, throughout the course of these LSSFEs, the MUFA contents of these oils increased for PUFA-rich soybean and corn oils from the 30 min time-point (a consequence of primary PUFA consumption), whereas those of the extra-virgin olive and avocado oils decreased from this time-point, which indicates the delayed onset of MUFA peroxidation, a process following primary PUFA oxidation. Moreover, the PUFA contents of the two soybean and single corn oil products decreased from 20 min, whereas those of both extra-virgin olive and the avocado oil products were lowered from the 30 min time-point. 

**Figure 2:**
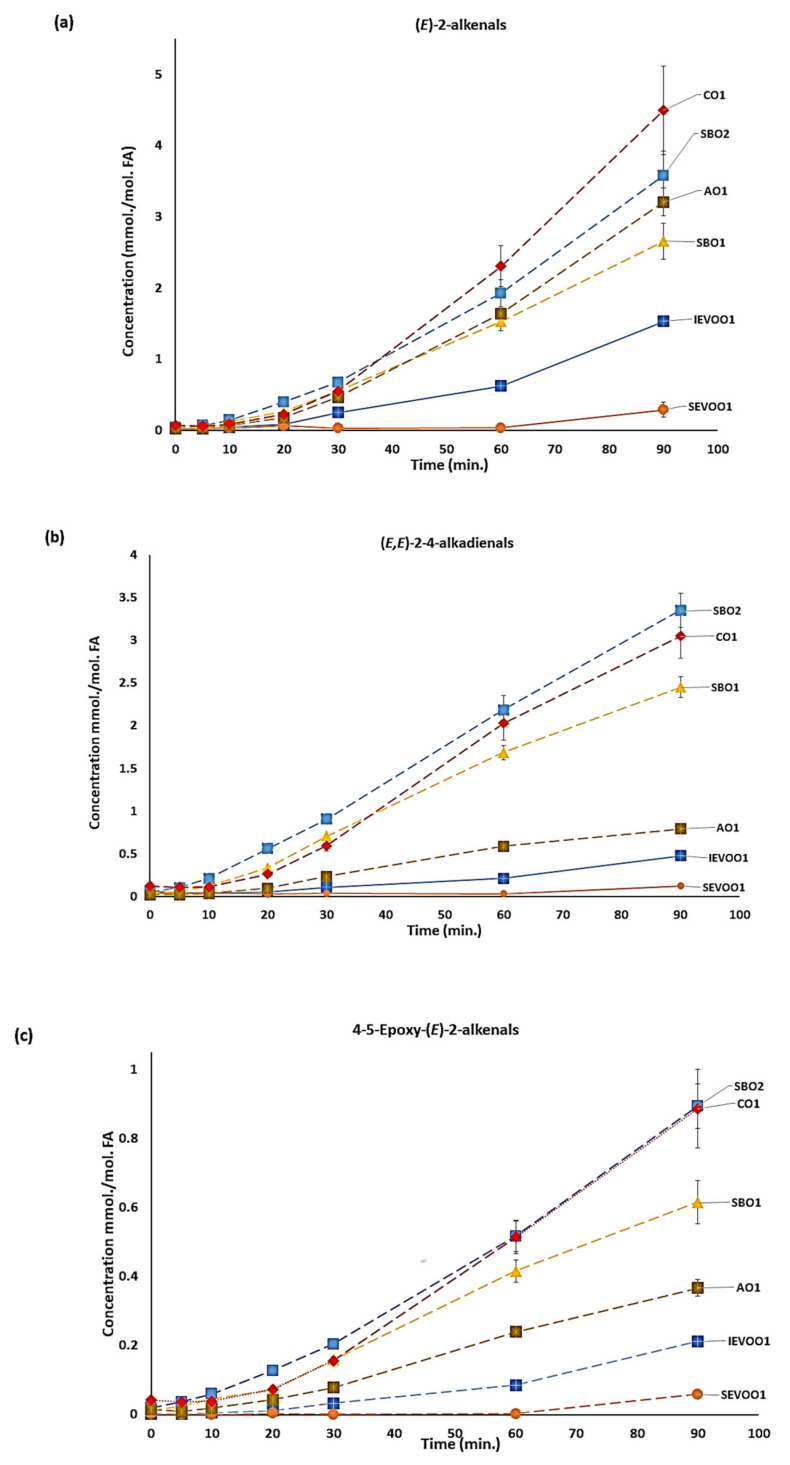

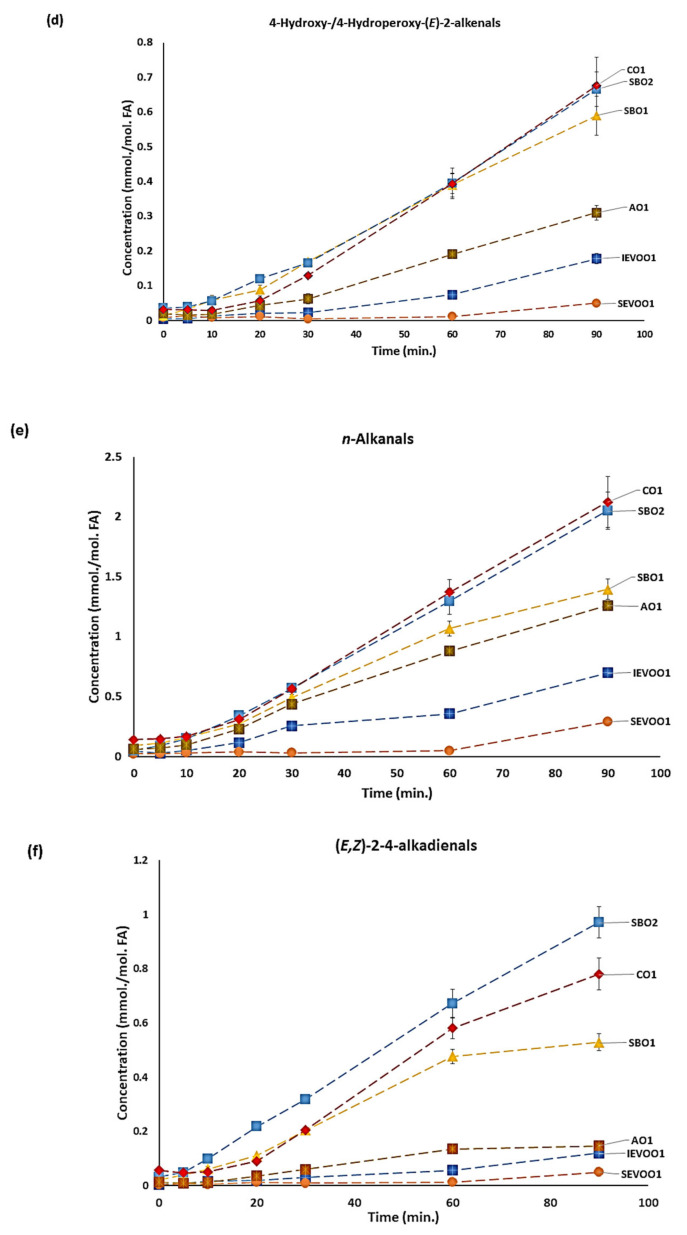

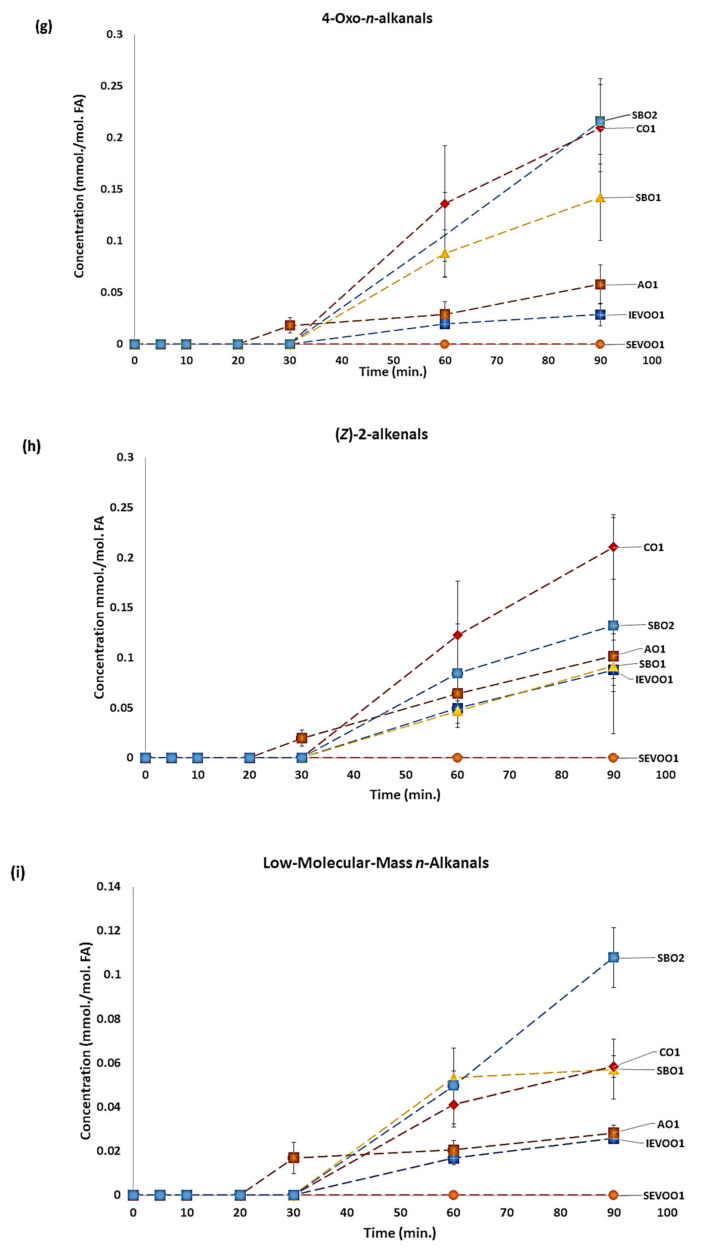
Plots of mean ± SEM ^1^H NMR-determined aldehyde concentrations (mmol./mol. FA) for culinary oils exposed to LSSFEs at 180^o^C for 0, 5, 10, 20, 30, 60 and 90 min durations (*n* = 3 replicate samples for each sampled time-point for each oil product). (**a**), (*E*)-2-Alkenals; (**b**), (*E,E*)-Alka-2,4-dienals; (**c**), 4-5-Epoxy-(*E*)-2-alkenals; (**d**), 4-Hydroxy-/4-Hydroperoxy-(*E*)-2-alkenals; (**e**), *n*-Alkanals; (**f**), (*E,Z*)-2-4-alkadienals; (**g**), 4-Oxo-*n*-alkanals; (**h**), (*Z*)-2-alkenals; (**i**), Low-Molecular-Mass *n*-Alkanals. Oil type abbreviations: as in Section 2.1.

As expected, the culinary oil x time-point first-order interaction effect was also extremely significant for all oils tested (*p* < 10^−50^ to <10^−18^), and this ratifies the contrasting responses of each oil type to increasing LSSFE lengths; for example, differential rates and extents PUFA losses from soybean/corn oils and extra-virgin olive/avocado oils (Figure S1b), and likewise the contrasting elevations and reductions in oil MUFA contents for these two groups respectively (Figure S1a). Our results also demonstrated that the ‘health-friendly’ ω-3 PUFAs, which are readily ^1^H NMR-detectable in soybean oil products, were particularly susceptible to thermal degradation during the LSSFE heating process, and therefore such vegetable and other oils marketed with the nutritional benefits offered by such FAs therein contain significantly diminished levels of these nutrients after being exposed to commercial or domestic frying or cooking processes. For these data, the specific ω-3 FA function resonances present in both soybean oil products tested showed reductions in the contents of linolenoylglycerols of 1.0 mol. % for SBO1, and 1.5 mol. % for SBO2 at the 90 min LSSFE sampling time-point (Supplementary Materials, Table S1 and Figure S1e). Furthermore, despite the presence of tocopherol antioxidants in such products [18], the realm of aldehydic LOPs generated remained in abundance (Figure 2). Indeed, these results corroborate with those of previous studies, which have indicated that antioxidants such as naturally present α-TOH, and added tocopherol esters and BHA, exhibit only very limited antioxidant properties at high frying temperatures [3,8,17]. Moreover, α-TOH has been shown to be thermally unstable at temperatures as low as 140 °C [19].

### 3.2. ^1^H NMR Monitoring of the Evolution of Secondary Aldehydic LOPs in Culinary Oils during Their Exposure to LSSFEs

All aldehyde species investigated are ^1^H NMR-detectable in the 9.00–10.20 ppm region of the spectral profiles obtained as their -CHO functional group protons, and individual aldehyde assignments and the multiplicities of resonances are highlighted in Table 3 as resonance assignment codes 1–10 (Figure 1b). These aldehyde signals were integrated relative to the total terminal-CH_3_ group resonances of total oil FAs, and their concentrations are expressed in mmol./mol. total FA units. From recent studies featuring determinations of the molecular structures of aldehydic LOPs derived from the peroxidation of plentiful ω-3 FAs in marine oils [15], the saturated nature of those giving rise to -CHO resonances located within the 9.70–9.90 ppm regions of spectra acquired on its thermo-oxidised samples (i.e., both high- and low-molecular-mass *n*-alkanals, and 4-oxo-*n*-alkanals), was confirmed by the acquisition of 2D ^1^H-^1^H COSY spectra. Indeed, all adjacent ^1^H nucleus correlations observed for resonances within this region were found to be localised in the δ = 2.37–2.80 ppm spectral sector, an observation confirming the presence of saturated methylene (-CH_2_-) groups at the α-position for all species involved. 

As expected, aldehyde formation increases with increasing thermal stressing period (Figure 2), and these values were also clearly critically dependent on oil nature and composition, with the highest levels of all aldehydes monitored being generated in PUFA-rich soybean and corn oils. Indeed, this time-dependent trend was observed across all aldehyde species, with the exception of (*E*)-2-alkenals, for which one of the soybean oil products tested (SBO1) generated slightly lower quantities at the 90 min time-point. However, (*E*)-2-Alkenals have also been shown to be produced as prominent secondary LOPs arising from the oxidation of oleoylglycerols in oil products rich in these FAs [1,16]. As noted from Figure 2, the relationship observed between aldehyde concentrations produced and LSSFE heating time is sigmoidal (i.e., S-shaped), since the lipid peroxidation process is an autocatalytic, self-propagating process, and is dependent on the primary formation and subsequent fragmentation of lipid hydroperoxides such as PUFA-derived CHPDs; a full list of mean concentration values for 9 of the aldehydes generated in each oil product at each LSSFE sampling time-point is provided in Table S2 of the Supplementary Materials. Prior to the 30 min time-point of LSSFE exposure, secondary aldehydic LOPs detectable in extra-virgin olive oil samples were present at only low quantities. However, this was not in the case for soybean and corn oils (Figure 2), with significant amounts being generated at this time-point. Indeed, ordering of the magnitudes of the levels of aldehydic LOPs generated in these oil products at this time-point only gave a ranking of SBO2 > SBO1 > CO1 > AVO1 > IEVOO1 > SEVOO1 for total α,β-unsaturated aldehydes, and SBO2 ≈ CO ≈ AVO1 > SBO1 > IEVOO1 > SEVOO1 for arguably less toxic *n*-alkanals (Table S3 of Supplementary Materials). These values are again reflective of the relative PUFA and MUFA contents of these oils, with the latter being much more resistant to peroxidation than the former. 

Notwithstanding, it appears that the greater susceptibilities of linolenoylglycerol species to peroxidation is responsible for the higher α,β-unsaturated aldehyde levels found in both soybean oil products at this quite low LSSFE time-point, i.e., a higher rate of CHPD generation and fragmentation to such aldehydes than that observed with linoleoylglycerols. In general, corn oil and one of the soybean oil products (SBO2) produced the highest levels of aldehydes throughout the thermal stressing time course at 180 °C. Although at the 90 min time-point, these levels were very similar for (*E,E*)-alka-2,4-dienals, 4,5-epoxy-(*E*)-2-alkenals, 4-hydroxy-/4-hydroperoxy-(*E*)-2-alkenals, *n*-alkanals. and 4-oxo-2-alkanals produced in these oils, concentrations of (*E*)-2-alkenals were somewhat greater in corn oil than in SBO2. However, the SBO2 product generated higher levels of LMM *n*-alkanals than both corn oil and the other soybean oil product (SBO1); these SBO2 product concentrations were also greater for (*E,Z*)-alka-2,4-dienals (Figure 2 and Table S3). The higher LMM *n*-alkanal concentrations, including propanal, generated in both SBO products, are consistent with their specific generation from their higher contents of ω-3 FA TAG species [1,6].

Although the source of 4-oxo-*n*-alkanals is not simply explicable from a full survey conducted on literature available, Dick et al. [20] found that 4-hydroxynonanal (HNA) arose from the actions of alkenal oxidoreductases on the linoleoylglycerol peroxidation product 4-hydroxy-(*E*)-2-nonenal (4-HNE) in vivo, and Kubatova et al. [21] noted that 4-oxo-*n*-nonanal, a highly potent lysyl alkylating agent, was a product generated in their experiments focused on the metabolism of HNE in central nervous system (CNS) models. Hence, this process appears to involve a sequentially mediated reduction of the (*E*)-2-nonenal chain to its saturated nonanal derivative via alkenal oxidoreductase activities, and the possible oxidation of its 4-position primary hydroxyl function through the actions of any available alcohol dehydrogenases [22]. However, since 4-oxo-(*E*)-nonenal (4-ONE) is a known lipid peroxidation product [22], it is feasible that a direct uncatalyzed chemical pathway involving the generation of 4-oxo-*n*-alkanals directly from reduction of their corresponding 4-oxo-(*E*)-2-alkenal species, i.e., 4-oxo-*n*-nonanal from linoleoylglycerol-derived 4-ONE, and 4-oxo-*n*-hexanal from linolenoylglycerol-derived 4-oxo-(*E*)-2-hexenal, occurs in culinary oils following prolonged LSSFE thermal stressing periods, i.e., those exposed for ≥60 min Interestingly, a correlation analysis of the complete dataset featuring all time-points revealed that there was a strong positive correlation between 4-oxo-*n*-alkanal and the combined 4-hydroxy/4-hydroperoxy-(*E*)-2-alkenal levels in mmol./mol total FA units (r = 0.89), as may be expected if this hypothesis was correct (4-hydroxy-(*E*)-2-alkenals arise from reduction of their corresponding hydroperoxy- derivatives [17]). 

Since 4-oxo-*n*-alkanals produced at relatively high levels in soybean oil products at LSSFE time-points of ≥60 min were also found to be strongly correlated with LMM *n*-alkanals (r = 0.93), and that both these aldehydic LOPs strongly loaded on the same PC in PCA analysis (Section 3.3), it appears that the 4-oxo-*n*-alkanal species monitored has a precursor that arises from the peroxidation of linolenoylglycerols, i.e., 4-oxo-(*E*)-hexenal, which potentially generates 4-oxo-*n*-hexanal at high temperatures. 

Univariate ANOVA of the time-dependent aldehyde level datasets obtained from all culinary oils investigated demonstrated that differences between oil product and sampling time-point (0–90 min) mean values were both very highly significantly different for all 9 aldehydes individually evaluated (*p* < 10^−6^ in each case). Individual differences between these values for the different oils at each time-point, and between the time-points themselves, are also clearly visible in the mean ± SEM plots shown in Figure 2; *post-hoc* Bonferroni-corrected two sample comparisons made between their least square mean (LSM) values are reported in Table S4 (Supplementary Materials). Notably, the culinary oil x time-point first-order interaction effect was also very highly significant for all aldehydes evaluated (*p* < 10^−6^ again), and this confirms the differential responses of aldehydic LOP concentration variables with respect to LSSFE heating time-point for each oil examined, as might be expected in view of their differing FA contents and hence differing patterns of LOPs generated (both primary and secondary), along with differing aldehydic LOP b.pts. This is also clearly apparent in Figure 2. With the exception of avocado oil, all of the oils tested did not evolve any ^1^H NMR-detectable 4-oxo-*n*-alkanals, LMM *n*-alkanals, nor (*Z*)-2-alkenals until the 60 min LSSFE time-point. 

From these *post-hoc* test results, it should be noted that soybean oil product SBO2 had least-squares mean (LSM) values that were not univariately significantly different from those of PUFA-rich corn oil for (*E*)-2-alkenals, 4,5-epoxy-(*E*)-2-alkenals, combined 4-hydroxy-/4-hydroperoxy-(*E*)-2-alkenals, *n*-alkanals, 4-oxo-*n*-alkanals and (*Z*)-2-alkenals. However, for both alka-2,4-dienal isomers (i.e., (*E,E*)- and (*E,Z*)-forms), which are PUFA- but not MUFA-derived secondary LOPs, significantly higher concentrations were found in the SBO2 product over that of corn oil. Moreover, as expected, in view of their relatively high ω-3 FA contents, both soybean oil products evaluated yielded significantly greater LMM *n*-alkanal levels than that of corn oil, notably at the 60 and 90 min LSSFE time-points (Table S4). For this LSM analysis, there was no significant difference observed between the two different soybean oils evaluated. Additionally, a UV statistical comparison of the ratios of LMM to high-molecular-mass *n*-alkanal LOP concentrations produced at the 90 min LSSFE time-point demonstrated that the values for soybean oils SBO1 and SBO2 (0.040 and 0.052, respectively) were indeed both significantly higher than that found for corn oil (0.027), *p* < 0.01 (ANOVA Welch test), and this again provided evidence that linolenoylglycerol species serve as substrates for the LMM homologues. 

As expected from previous investigations [1], (*E*)-2-alkenals were only marginally generated up until the 90 min heating period in extra-virgin olive oils (Figure 2), most notably in the Spanish product. Conversely, corn oil produced the highest level of this class of α,β-unsaturated aldehydes, with soybean and avocado oils also generating relatively high concentrations.

### 3.3. Multivariate Chemometric Analyses of Product-Dependent ^1^H NMR-Detectable Aldehydic LOP Concentration Signatures Found in Thermally Stressed Culinary Oil Products

**Figure 3:**
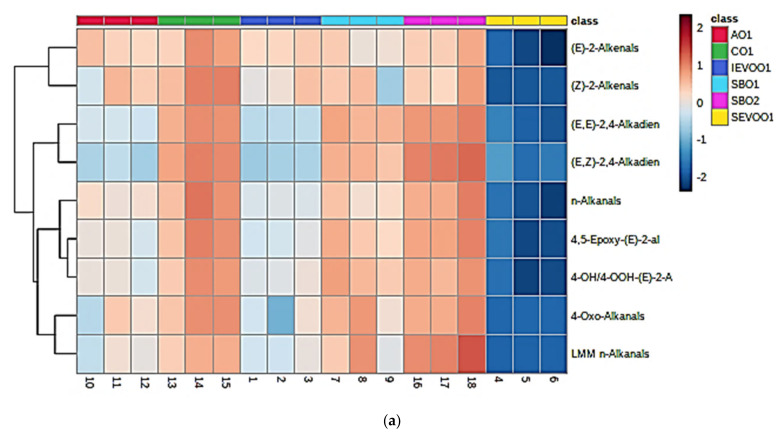

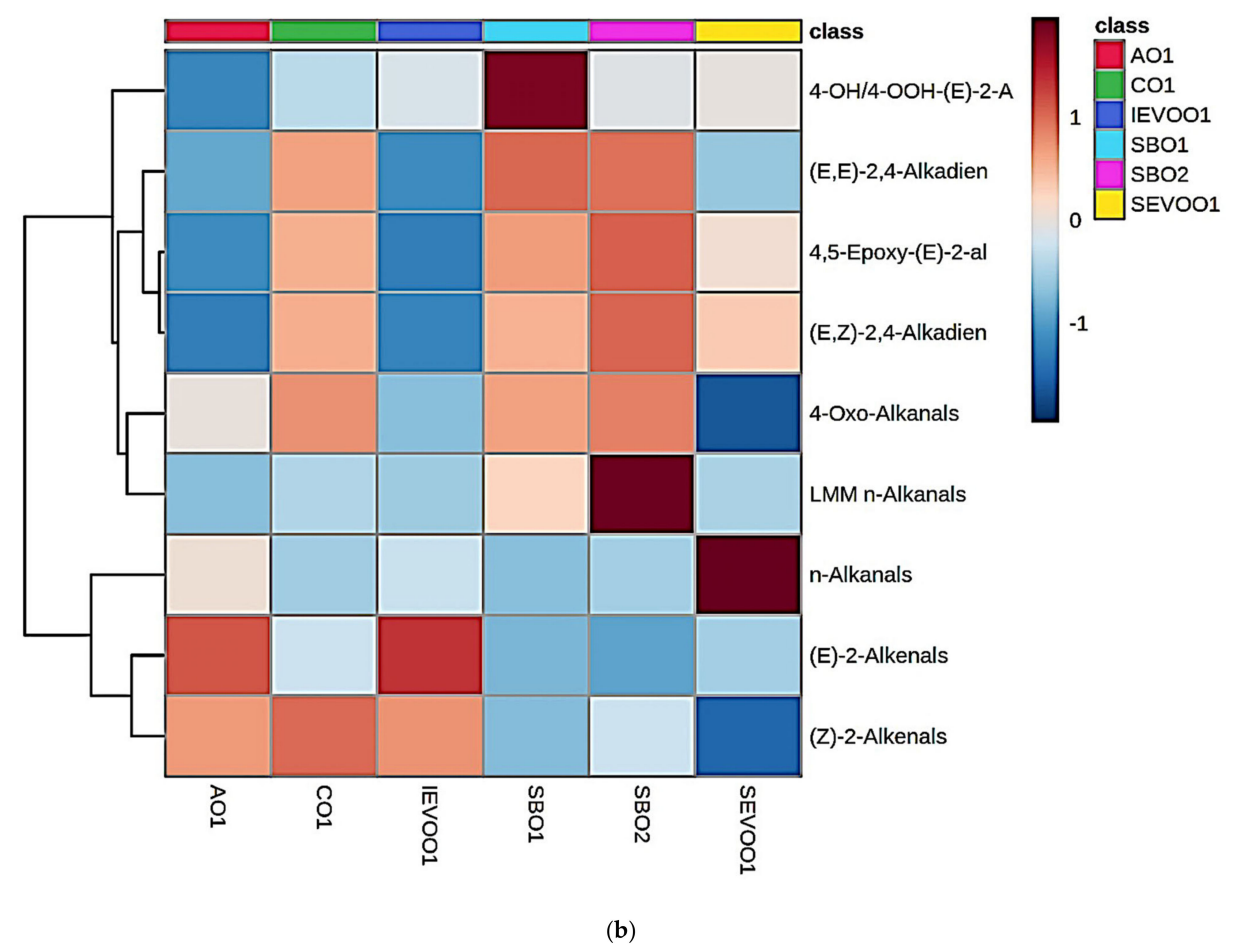
(**a**) Heatmap diagram displaying the nature, extent and ANOVA-based significance of univariate differences between all nine aldehydic LOP variables (near right-hand side ordinate axis) for the avocado (red), corn (green), Italian extra-virgin olive (dark blue), soybean 1 (cyan), soybean 2 (mauve) and Spanish extra-virgin olive 1 (yellow) oil products at the 90 min LSSFE time-point only. The complete dataset was glog-transformed and autoscaled prior to analysis, but not constant sum-normalized (CSN). Transformed analyte intensities are shown in the far right-hand side ordinate axis: deep blue and red colorations represent extremes of low and high concentrations, respectively. The left-hand side ordinate axis of this plot shows results derived from an associated agglomerative hierarchical clustering (AHC) analysis of these aldehyde variables. (**b**) Corresponding heatmap diagram of only the 90 min heating time-point dataset, but following the application of CSN prior to analysis. Oil type abbreviations: as Section 2.1.

Figure 3a shows ANOVA-based heatmaps of all aldehyde levels determined at the 90 min time-point only, and in addition to clearly demonstrating the much higher levels of these secondary LOPs generated in the two soybean and the single corn oil products examined, this also provides some evidence for product-distinctive patterns of these toxins, for example higher levels of LMM- and 4-oxo-*n*-alkanals, the former known to arise from fragmentation of CHPDs generated from the primary peroxidation of ω-3 FAs in soybean oils [1,6]. The associated AHC analysis performed revealed two major analyte clusterings, with four sub-clusterings for one of these. The top left-hand side major cluster comprised (*E*)-2- and (*Z*)-2-alkenals only, which are expected to be associated since they are both PUFA-derived secondary LOPs [23,24], and the latter species has been postulated to arise from thermal isomerism of the former [1,25]; (*E*)-2-alkenals may, however, also be generated from the peroxidation of MUFA substrates. Notwithstanding, the second major cluster contains all other aldehyde analyte variables. The downwards first (1), second (2), third (3) and fourth (4) sub-clusters within the bottom left-hand side major cluster feature (1) both (*E,E*)- and (*E.Z*)-alka-2,4-dienal isomers, which again could be related in view of a possible thermal isomeric interconversion process [1]; (2) *n*-alkanals alone, which are derived the peroxidation of both PUFA and MUFA sources; (3) 4,5-epoxy- and 4-hydroxy-/4-hydroperoxy-(*E*)-2-alkenals, which both arise from PUFA peroxidation, the former from their corresponding (*E.E*)-alka-2,4-dienal precursors; and (4) LMM- and 4-oxo-*n*-alkanals, which presumably both arise from the peroxidation of ω-3 PUFAs in view of their strong positive correlation, as noted above. 

However, it should also be noted that 2-heptenal isomers may be generated from alka-2,4-decadienal decomposition, together with acrolein, hexanal, acetaldehyde, butenal, 2-heptenal, 2-octenal, glyoxal, (*E*)-2-buten-1,4-dial and benzaldehyde [26]. Furthermore, alka-2,4-dienals may degrade to 2,3- or 4,5-epoxyaldehydes, which are then further degenerated to composites of either isomeric 2-octenals and acetaldehyde, or glyoxal and 2-octene [27,28]. 

Figure 3b, however, shows a corresponding heatmap of mean replicate aldehyde levels, in this case performed following the application of constant sum normalisation (CSN) in order to permit comparisons of proportionate contributions of aldehyde levels towards the total observed within each oil product studied, i.e., for the purpose of recognising any oil-dependent characteristic patterns of these LOPs. Indeed, this heatmap confirmed that at the 90 min LSSFE time-point, the soybean oil products contained proportionately higher levels of both (*E,E*)- and (*E,Z*)-alka-2,4-dienals, and 4,5-epoxy-(*E*)-2-alkenals, the latter being derived from (*E,E*)-alka-2,4-dienals [17]. Moreover, LMM *n*-alkanals were proportionately present at greater concentrations in both soybean oil products over those of all other oils investigated, and this is attributable to these oils’ considerably higher ω-3 PUFA contents. Additionally, one of the soybean oil products (SBO1) had very high proportionate levels of the 4-hydroxy-/4-hydroperoxy-(*E*)-2-alkenal classification, although both soybean oil products contained similar proportionate concentrations of this class of aldehydes to those of the corn oil samples analysed. Similarly, the AHC analysis arising therefrom was found to be similar to that obtained without the use of CSN. Indeed, these clustering features again corresponded to 4,5-epoxy-(*E*)-2-alkenals being derived from (*E,E*)-alka-2,4-dienals [17], ω-3 FA-sourced LMM *n*-alkanals being associated with 4-oxo-*n*-alkanals, high-molecular-mass *n*-alkanals and (*E*)-2-alkenals originating from both MUFA and PUFA sources, and (*Z*)-2-alkenals arising from the thermally induced isomerisation of their corresponding (*E*)-forms [1]. 

Subsequently, PCA was performed on the ^1^H NMR-determined aldehydic LOP dataset, i.e., all 9 aldehydes monitored, and this included all 0–90 min sampling time-points involved for each of the 6 culinary oil products exposed to LSSFEs at 180 °C. The optimal model for this MV analysis, which was developed with Varimax rotation and Kaiser normalisation, contained 3 orthogonal factors (principal components, PCs, Table 4). Of the 3 PCs isolable, the first, second and third accounted for 40.3, 32.2 and 25.6% of the total variance respectively (the fourth PC accounted for only 1.2%); these approximately corresponded to PC-loadings of 4, 3 and 2 aldehydic LOPs, respectively. Although, as expected, some aldehyde classifications loaded significantly on 2 or even all 3 PCs (detailed below), the strongest loadings for PC1 were found for (*E,E*)- and (*E.Z*)-2,4-alkadienals, combined 4-hydroxy-/4-hydroperoxy-(*E*)-2-alkenals, and 4,5-epoxy-(*E*)-2-alkenals; (*E*)- and (*Z*)-2-alkenals, and higher-molecular-mass *n*-alkanals on PC2; and 4-oxo-*n*-alkanals and LMM *n*-alkanals on PC3. For PC1, these observations are again explicable by the reported route of generation of 4,5-epoxy-(*E*)-2-alkenals from (*E.E*)-2,4-alkadienals [17], the possible thermal isomerism of this di-unsaturated aldehyde to its corresponding (*E,Z*)- form [1,25], and presumably also a common linoleoylglycerol source for all PC1-loading aldehydes featured, including the 4-hydroxy-/4-hydroperoxy-(*E*)-2-alkenal classification, in which case they would be present as nonenal homologues. For PC2, both (*E*)-2-alkenals and *n*-alkanals arise from the peroxidation of MUFAs as well as PUFAs, and (*Z*)-2-alkenals are generated from the thermal isomerism of their (*E*)-derivatives. Moreover, for PC3, LMM *n*-alkanals arise from peroxidised linolenoylglycerol sources, and arguably also 4-oxo-*n*-alkanal (as 4-oxo-*n*-hexanal), as considered above. 

Hence, despite at least some aldehydes exerting a significant loading vector impact on PCs other than that upon which they loaded the most strongly, it appears that those loading the strongest on PCs 1, 2 and 3 may originally arise from common sources, and these results are consistent with linoleoylglycerol, oleoylglycerol and linolenoylglycerol origins respectively, although the thermally-induced isomerism of selected aldehyde classes, and the further degradation of more structurally complex ones to simpler species [1], clearly complicates this form of analysis and its interpretation. Such co-loadings of aldehyde predictor variables on up to two other PCs are explicable by (1) the differential UFA sources of differing homologues of the same classes of aldehydes, (e.g., (*E*)-2-alkenals arising from the fragmentation of either CHPDs or HPMs, which are, in turn, derived from the peroxidation of PUFAs and MUFAs, respectively, and 4-hydroxy-(*E*)-2-alkenals derived from either linoleoyl- or linolenoylglycerol sources, i.e., 4-hydroxy-(*E*)-2-nonenal and -hexenal form the former, and 4-hydroxy-(*E*)-2-heptenal and -pentenal from the latter); and (2) differential mechanisms available for their post-generation isomerism and/or decomposition. 

**Figure 4:**
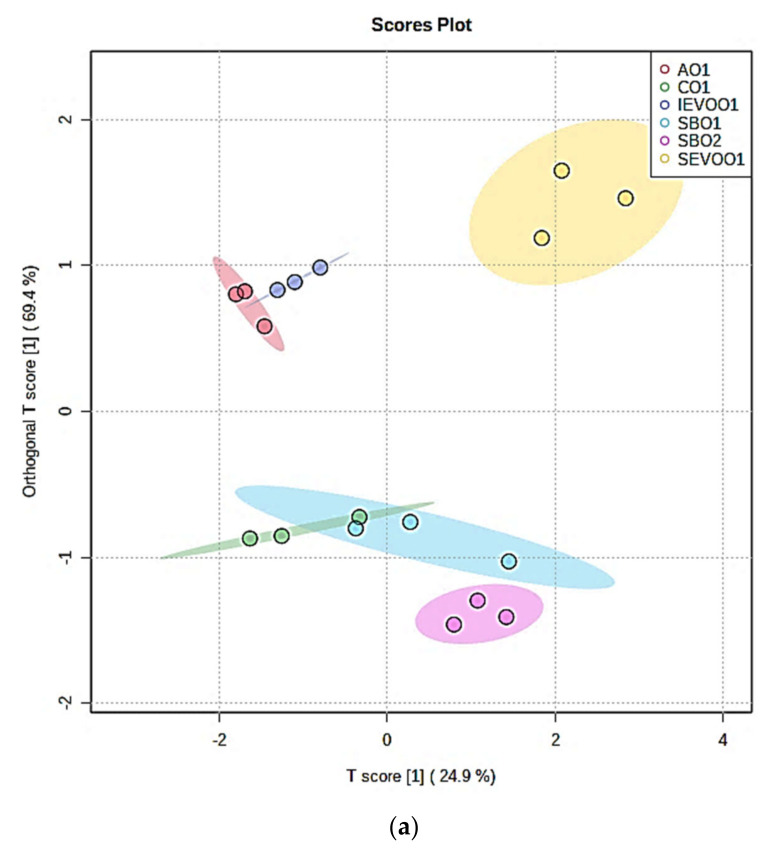

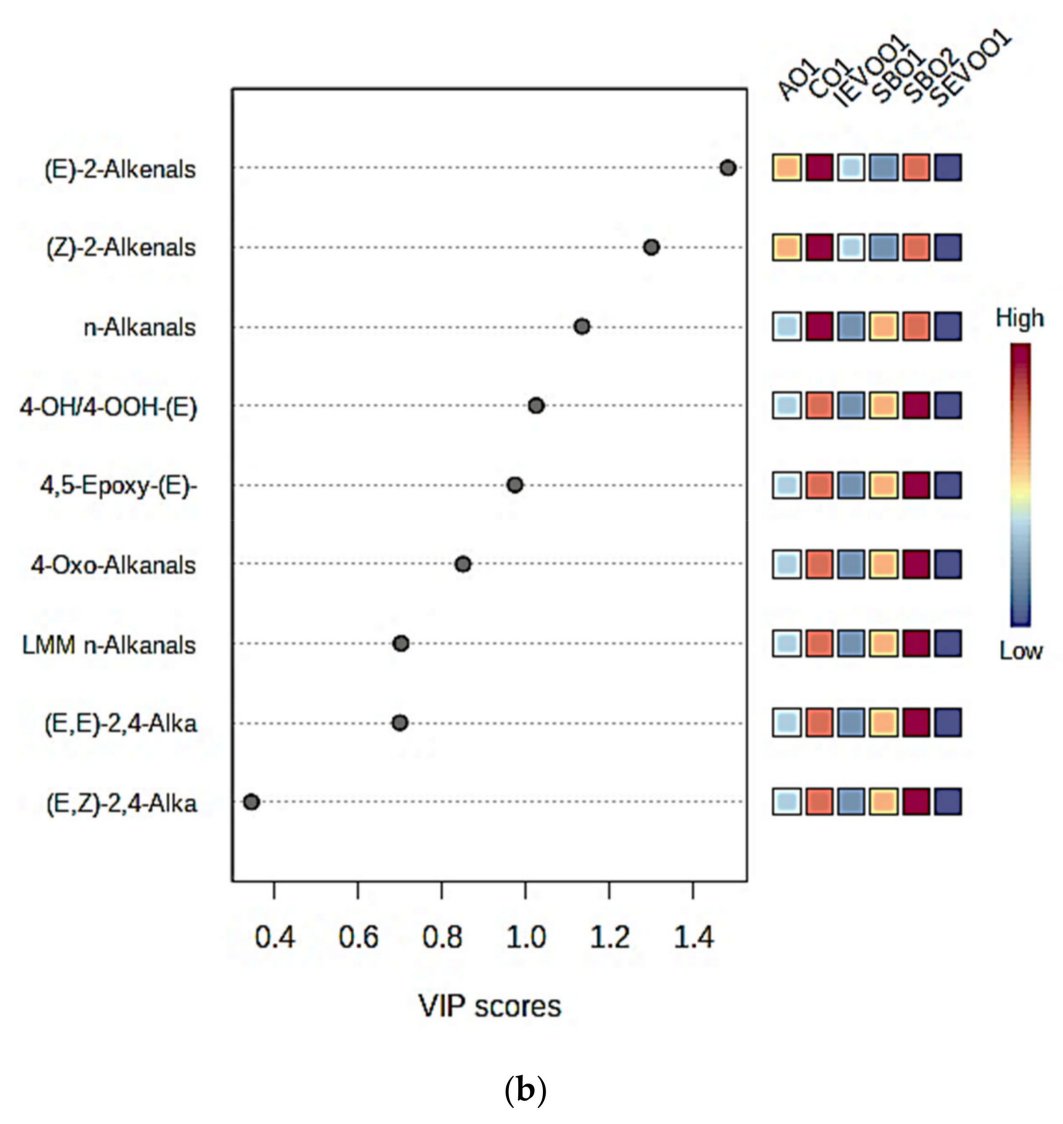
(**a**) OPLS-DA scores plot of orthogonal T score [1] versus T score [1], showing distinctions between patterns of aldehydic LOP concentrations generated in the oils tested at the 90 min LSSFE time-point. (**b**) Variable importance parameter (VIP) values for the OPLS-DA model applied in (**a**). Abbreviations, as in Section 2.1, with 4-OH/4-OOH-(E), 4,5-Epoxy-(E)-, (E,E)-2,4-Alka and (E,Z)-2,4-Alka representing combined 4-hydroxy-/4-hydroperoxy-(*E*)-2-alkenals, 4,5-epoxy-(*E*)-2-alkenals, (*E,E*)-2,4-alkadienals and (*E,Z*)-2,4-alkadienals, respectively.

For the 90 min LSSFE time-point dataset, both PLS-DA and OPLS-DA scores plots demonstrated a high level of discriminatory power between each oil type investigated (as shown for the latter in Figure 4a). Indeed, although there was a small level of overlap between the CO1 and SBO1 sample clusters, aldehyde patterns arising from both soybean oil products remained distinct from all the other culinary oils evaluated. For this discrimination, cross-validation Q^2^ and R^2^Y values obtained for the OPLS-DA model were 0.73 and 0.85, respectively (permutation testing *p* value <5.0 × 10^−4^), and those for the PLS-DA model were 0.67 and 0.86 respectively (*p* = 0.007). Considering that there were six different culinary oils for comparison, these values are indeed excellent, and demonstrate a high level of discriminatory potential for both MV analysis techniques employed. Variable importance parameters (VIPs) for the aldehyde predictor variables (Figure 4b) were in the order (*E*)-2-alkenals > (*Z*)-2-alkenals > *n*-alkanals > 4-hydroxy-/4-hydroperoxy-(*E*)-2-alkenals > 4,5-epoxy-(*E*)-2-alkenals (VIP values all >1), followed by 4-oxo-*n*-alkanals > LMM *n*-alkanals > (*E,E*)-alka-2,4-dienals (VIP values 0.70–0.92). Since (*E,Z*)-alka-2,4-dienals had a VIP parameter of only <0.40, its overall discriminatory power was of little or no value. 

However, one cautionary note and limitation of the work performed in the current study is that the ω-3 PUFA peroxidation product propanal has a very low b.pt (only 49 °C) [2], and therefore levels monitored in thermally stressed culinary oil products, particularly soybean oils, are likely to represent only a small or very small proportion of the total generated from their exposure to the thermo-oxidative LSSFEs featured. In point of fact, our solution-state ^1^H NMR studies only detect and determine the amounts of aldehydic LOPs which are remaining in these oil products following their exposure to high-temperature LSSFEs, a process which clearly gives rise to the vaporisation of many of them, most especially those with b.pts which lie some way below that of the thermal stressing temperature employed (180 °C), as reviewed in [2]. Nevertheless, these ‘oil solution’ forms of aldehydes largely represent the fractions of these toxins which are transferable to fried foods during such thermo-oxidative episodes, and arguably also those which are ingestible by humans when consuming fried foods such as potato chips [1,2]. As expected, potato chip levels of nascent aldehydic LOP toxins appear to be linearly proportional to the mass of fryer-used culinary oils taken up by these commonly consumed foods throughout the frying process [1].

### 3.4. Overview of Experimental Results Obtained

One major objective of the current study was to conduct both UV and MV ^1^H NMR-based chemometrics analysis in order to explore the acylglycerol FA sources of aldehydic LOPs, and employ ‘state-of-the-art’ chemometrics strategies to distinguish between the signatures of aldehydic LOPs generated in thermally stressed ω-3 FA-containing soybean oils from those yielded from the thermo-oxidation of other culinary frying oils, including an ω-3 FA-deplete culinary oil with a similar total PUFA content (corn oil). 

Whereas ω-6 and ω-3 FAs are both present in in natural and refined soybean oil products, the major ω-3 FA therein, i.e., linolenic acid, thermo-oxidatively deteriorates more rapidly, and to a greater extent than ω-6 FAs. Moreover, the homologue status and patterns of different classes of LOPs generated therefrom, notably aldehydes, are clearly distinct from those arising from the peroxidation of ω-6 FAs [8.9]. Hence, the breakdown of these highly peroxidatively-susceptible FAs in soybean oils during their exposure to standard frying practices was expected to give rise to a differential pattern and higher levels of aldehydic LOPs when compared to those derived from ω-6 FAs (predominantly linoleic acid) on a molar equivalent basis [1,8]. 

Herein, we found that the relatively higher ω-3 FA contents of the soybean oil products tested generated higher levels of LMM *n*-alkanal LOPs over those of all other oils tested, although this observation was more pronounced for one of the two products investigated here (SBO2, Figure 2). Since both these oil products have very similar FA contents, this observation may be ascribable to higher levels of chain-breaking antioxidants present in the more peroxidation-resistant brand (SBO1), which was manufactured by an expeller-pressing process, and therefore may be expected to contain larger amounts of these protective agents. However, levels of both α- and ω-tocopherols in refined soybean oils are sub-millimolar (Section 3.6), and the markedly lower concentration of the former has indicated that such oil products may not represent an acceptable source of dietary vitamin E [29]. Nevertheless, such antioxidant levels may indeed offer at least some health and storage (shelf-life longevity) benefits to consumers.

Overall, when the PUFA and MUFA contents of cooking oils are higher and lower respectively in culinary oil products, greater levels of aldehydes are produced. As anticipated, all aldehydes derived from PUFA peroxidation alone, along with those arising from both MUFA and PUFA degeneration such as (*E*)-2-alkenals, were increased in concentration with increasing oil PUFA content in view of their more rapid rate of peroxidation over that of MUFAs, and also an increased rate of decomposition of primary CHPDs than that of HPM peroxidation products. 

However, 2-alkenals and alka-2,4-dienals have also been shown to thermally degrade at a temperature of 200 °C. Indeed, Zamora et al. [30] explored the thermally-induced decomposition of 2-pentenal and -octenal, and 2,4-heptadienal and 2,4-decadienal, in order to determine their stability at this high temperature, and also to identify and monitor the levels of products arising therefrom. Results acquired demonstrated that both these classes of aldehydes rapidly degraded in the presence of air and an aqueous buffering system, generating formaldehyde and acetaldehyde, along with products arising from >CH=CH< bond cleavage: specifically, propanal and hexanal from 2-pentenal and 2-octenal, respectively, and propanal/2-pentenal and hexanal/2-octenal admixtures from 2,4-heptadienal and 2,4-decadienal, respectively (glyoxal and fumaraldehyde were also significant degradation products). Therefore, propanal, which arises as a secondary LOP from the peroxidation of linolenoylglycerol substrates, may also be yielded from the thermally induced decomposition of either 2-pentenal or 2,4-heptadienal. Fortunately, both these aldehydes are also derived from ω-3 FA peroxidation [15,31,32]. For the studies reported in Ref. [30], the activation energy of >CH=CH< double bond cleavage was found to be quite low (∼25 kJ/mol), and overall, the levels of alkanals generated (10–18%) were greater than that of 2-alkenals (∼1%). These results are comparable to those reported in references [27] and [28]. 

Although the above thermally-induced decompositions of higher homologue α,β-unsaturated aldehydes to lower ones is promoted by exposure to atmospheric O_2_, and the presence of an aqueous buffering system, it should be noted that refined, unused culinary vegetable oils usually contains a water content of approximately 0.1% (*w*/*w*), and more importantly used frying oil contains 0.3–1.5% (*w*/*w*) of it at standard frying temperatures [33]. Hence, it is anticipated that culinary oil water may strongly facilitate these reactions during standard frying practices, most especially in water-rich foods fried in such oil media. 

^1^H NMR analysis performed at an operating frequency of 600 MHz can simultaneously identify and quantify a range of aldehydic LOPs (up to 10 noted in this study), and therefore provides a greater overall evaluation of the oxidative deterioration status of oils exposed to standard or unusual frying practices than that conducted at lower field strengths, e.g., at only 400 MHz. Moreover, high-resolution NMR spectroscopy can successfully resolve resonances arising from different geometric (configurational) isomers of different classes of α,β-unsaturated aldehydes, and has the ability to simultaneously determine the nature and level of UFA acyl chain degradations (Table S1 and Figure S1).

### 3.5. Discussion of Experimental Design and Statistical Analysis of the Results Acquired 

For the investigation of distinctive patterns of aldehydic LOPs thermo-oxidatively generated in differing culinary oils during their exposure to LSSFEs, including that arising from the distinctively higher ω-3 FA content of soybean oils, from those mainly derived from linoleoylglycerol and/or oleoylglycerol peroxidation in linolenoylglycerol-depelete oils (e.g., corn oil), we employed a combination of both UV and MV statistical analysis techniques. This was considered essential, since some analyte variables may be univariately significant, although not multivariately so; indeed, this is a quite frequent observation in chemometrics studies, which may occur from the ability of such a variable to serve as a singular, orthogonal marker which remains unassociated (or uncorrelated) with others, i.e., other aldehydes such as those monitored in this study. Conversely, although a selected potential marker analyte may not prove to be univariately significant, it may indeed linger as one being reasonably or even strongly correlated to other analytes, which together comprise a powerful MV pattern (orthogonal component), which may indeed be useful for distinguishing between two pre-defined, comparative groups of samples. Currently the majority of chemometrics-based researchers much prefer to conduct a composite of both these forms of statistical analyses on datasets investigated, although for the UV approach, recommended corrections to significance levels should be applied. Since these issues are beyond the scope of this paper, readers are referred to references [34,35] for further information. 

### 3.6. Deleterious Health Effects Potentially Arising from the Ingestion or Inhalation of Toxic Aldehydic LOPs, and Strategies for Their Circumvention in Soybean Frying Oils

In this investigation, high-resolution ^1^H NMR analysis has established that higher levels of aldehydes are observed in PUFA-rich rather than MUFA-rich oils, as expected [36,37,38]. Moreover, the pattern of aldehydic LOPs generated in soybean oils was found to differ significantly from that of corn oil, which has a similar total PUFA, but much lower ω-3 FA content. The deleterious health risks presented by the ingestion of high concentrations of aldehydes, e.g., those present in fried foods such as French fries (which contain up to 30% (*w*/*w*) or even higher quantities of LOP-containing pre-heated oils [39]), or alternatively directly inhaled in their vapourised forms, afford significant concerns to both consumers and restaurant employees respectively.

Studies dating back over the past 25 years or so [1,2,3,39,40,41] have shown that the ingestion of dietary aldehydic LOPs is associated with the induction, development and/or perpetuation of deleterious health effects in humans dietarily ingesting them in foods, particularly fried foods, and these include neurodegenerative disorders [42], atherosclerosis and its cardiovascular sequelae [43,44], cancer [45] and liver damage [46], for example, together with a range of further non-communicable diseases [34]. Globally, cardiovascular diseases (CVDs) have remained the leading cause of death in humans for the past 15 years [47]. Moreover, fried food intakes, which are known to contain aldehydic LOPs [1,39], have been correlated with all-cause, cancer and CVD mortalities in a recent prospective cohort study [48]. Cytotoxic and genotoxic aldehydes generated from culinary frying or cooking practices have previously been shown to be absorbed from the gut into the systemic circulation in vivo [40], where they may gain access to critical organs and tissues which potentially serve as toxicological targets. Observations within eukaryotic models have also observed that aldehydes cause oxidative stress damage to cells, and chemical modification of DNA [49]. Indeed, the biomolecularly-destructive effects of aldehyde species have been demonstrated in animal models as histopathological modifications of the liver, colon and jejunum [46], and that report confirmed that these essential organs were damaged following the oral administration of pre-heated, PUFA-rich cooking oils, but not of corresponding unheated products. Moreover, once absorbed into the systemic circulation, these aldehydes have the ability to propagate foam cell formation from macrophages, a critical stage in the pathogeneses of atherosclerosis [40,44]. Furthermore, it has been demonstrated in a rat model that the ingestion of LOPs administered as thermally-stressed safflower oil markedly enhanced the occurrence of malformations in embryos [50], i.e., they exert teratogenic properties. 

Of much interest, 4-ONE, the unsaturated derivative of 4-oxo-(*E*)-nonanal, and generated in thermally stressed soybean oils (putatively as the 4-oxo-(*E*)-hexanal homologue from peroxidised linolenoylglycerols), has been found to exert a higher level of toxicity than the much more highly studied 4-HNE in a number of experimental models. For example, data available in reference [51] revealed that 4-ONE potently alters mitochondrial function by acting as an effector of lipid peroxidation within this environment. 

Intriguingly, the inhalation of vapourised aldehydes generated during high-temperature frying or wok-cooking practices has been correlated with a higher prevalence of bronchitis in epidemiological studies [52]. Indeed, it was found that women aged 20–40 who had been exposed to cooking oil fumes >21 times per week had a >4-fold higher risk of developing chronic bronchitis over those who were exposed <14 times a week. Epidemiological studies performed on non-smoking Taiwanese women have demonstrated an increased incidence of lung cancer amongst those who cooked more meals per day [53]. Intriguingly, it was also noted that the risk of lung cancer was higher if women waited until visible fumes were produced, prior to cooking episodes [53]. Deaths attributable to lung cancer have also been ranked as a leading cause of mortality worldwide (World Health Organisation 2016) [47], and this study therefore provided a further understanding of alternative, non-tobacco smoking, causes of this condition. Notably, in 2016, soybean oil was rated the most commonly consumed vegetable oil in China (44%), with rapeseed, palm and peanut oils rating second, third and fourth (24, 18 and 9%), respectively [54]. Elevated levels of urinary oxidative stress biomarkers, specifically 1-hydroxypyrene, malondialdehyde (MDA) and 8-hydroxy-2′-deoxyguanosine, were observed in restaurant workers routinely exposed to cooking oil fumes [55,56]; MDA presumably at least partially arises from cooking oil fume ingestion, since this aldehyde is a well-known cytotoxic and genotoxic LOP arising from the oxidation of ω-3 FAs [2]. Indeed, a significant proportion of aldehydic LOPs have boiling-points (b.pts) close to, below or much below those of standard frying temperatures (*ca.* 180 °C) [2]. In 2013, Lee and Gany [57] conducted a literature review of the risks of lung cancer associated with cooking oil fume inhalation, and they found that all investigations which explored the mechanisms associated with these increased risks uncovered evidence for mutagens and/or carcinogens in cooking oil fume extracts, and/or relevant molecular routes for DNA damage or carcinogenesis. These researchers therefore strongly concluded that further studies should be performed in order to further explore at-risk groups based in the USA, along with the development and validation of powerful interventions focused on the curtailment of these risks. 

From the results acquired in the current study, PUFA-rich oils, notably soybean and corn oils, should not be recommended for high-temperature frying practices in view of their high susceptibilities to peroxidative degradation. Indeed, we recommend that MUFA-rich oils, including extra-virgin olive and avocado oils, should be utilised for industrial, restaurant-based and domestic frying practices instead because of their relatively high resistance against thermally-induced peroxidation. 

Evidence presented in the current study suggests that the employment of genetically engineered, high oleoylglycerol content soybean oils would be more suitable for high-temperature culinary frying or cooking purposes, in view of substantial reductions in PUFA content and consequently also those of toxic aldehydes formed during such frying episodes. By silencing the GmFAD3 gene, both linolenic acid and SFA contents can be reduced in soybean oil, a process which increases its stability and resistance to thermally- induced oxidation [58]. Alternatively, in order to prevent aldehyde formation, changes in cooking practices, including the restriction of O_2_ from the system, for example, via the instigation of vacuum frying techniques, would also serve to markedly reduce the formation of aldehydes and other LOPs during the thermal stressing of such frying oils. This methodology has been shown to reduce the contents of acrylamide in potato crisps by 98%, and also lowered polymeric TAG species in culinary oils evaluated [59]. Results obtained by Albertos et al. [60] corroborate the benefits of vacuum frying practices, including the diminished degradation of ω-3 FAs and tocopherols, as well as reduced concentrations of lipid hydroperoxides and their carbonyl fragmentation products. In view of the lower temperatures used during these cooking practices, many ’health-friendly’ agents, including chain-breaking antioxidants and ω-3 FAs themselves remain relatively thermally stable. A further approach is pressure frying, a strategy which has been adopted for the preparation of fried chicken products in numerous commercial restaurants focused on such fast-food commodities [61]. However, the cost and required user-knowledge and familiarity with these frying techniques represent two factors which may render their instigation and common use unattractive to fast-food vendors, especially those managing small, non-global restaurants. 

Another alternative involves the blending of two oils, for example one high in PUFAs, such as soybean oil, and others high in MUFAs such as camellia oil, in order to reduce lipid peroxidation and hence enhance frying stability. This has been explored when these two oils underwent deep-frying events, and results acquired showed that the peroxide and *p*-anisidine values of such blends were diminished significantly with higher camellia:soybean oil ratios [62]. 

An additional strategy features the supplementation of soybean oil products with dietary lipid-soluble, peroxyl radical-scavenging chain-breaking antioxidants. However, this vegetable oil already contains naturally occurring tocopherols (tocopherols and tocotrienols are present in different classes and sources of vegetable oils at a range of concentrations [29]). The antioxidant effectiveness of these agents depends on their derivative (aromatic methyl substituent positional) forms, and their relative activities in this context are, of course, concentration-dependent [63]. Typically, soybean oil contains higher and lower levels of the γ- and α-tocopherol forms, respectively, than those found in other PUFA-rich oils such as sunflower and rapeseed oils, although these levels are comparable to those found in corn oil [63]. However, previous investigations have demonstrated that tocopherols and other phenolic antioxidants are only poorly protective against the peroxidation of PUFAs taking place during high-temperature frying episodes, a consequence of (1) their inabilities to combat the vast thermally induced peroxidative cascade of autocatalytic free radical reactions occurring therein, especially when present at relatively low concentrations, which are insufficient to counter these effects, and (2) their thermal instabilities at these temperatures [3,19,40]. Moreover, tocopherols have b.pts which are not much greater than that of standard frying temperature [40], and therefore significant evaporative losses of these antioxidants can result from their exposure to standard frying temperatures. Alpha- and γ-tocopherol levels present in soybean oil have been reported as 71 ± 6 and 273 ± 11 mg/kg, i.e., 0.13 and 0.51 mmol./kg, respectively [29], values which are notably lower than those of other cooking oils, and which from the results acquired in the current study, are clearly insufficient to retard the peroxidation process during high-temperature frying practices. 

Nevertheless, polyphenols, which are powerful chain-breaking antioxidants, have been shown to offer strong protective activities against the thermo-oxidation of culinary oil PUFAs [64], and also suppress the thermal degradation of α-tocopherol at temperatures of 100–120 °C [65], and therefore supplementation of natural soybean oil samples with such agents may indeed serve to enhance the their safety margins when employed for domestic or commercial frying operations, which are usually conducted at 160–190 °C, although for standard frying practices it is 180 °C [30]. Indeed, using the Rancimat technique for evaluating the oxidative stability of culinary oils, it has been revealed that supplementation of edible vegetable oils with a ‘spectrum’ of polyphenols contained within an olive leaf extract increased the oil deterioration induction time from 19–54% [64], the highest increase being observed with sunflower oil, for which a ‘protection factor’ of 1.54 was recorded. 

Although quite rich in tocopherols, vegetable seed oils frequently employed for high-temperature frying purposes are, unfortunately, almost completely devoid of other phenolic antioxidant compounds, and therefore their supplementation with such polyphenolic antioxidants, for example those present in olive leaf extracts [64], may indeed be valuable for the protection of UFAs therein against adventitious peroxidation during periods of storage; such an approach may even offer some level of protection of UFA-rich frying oils when exposed to high-temperature frying episodes. However, olive oil embodies both tocopherols and phenolic compounds as antioxidants, especially the extra-virgin forms which are mainly used as salad oils. Hence, in addition to their high MUFA contents, phenolic and polyphenolic antioxidants contained within the two extra-virgin olive oil products investigated in the current study may also serve to enhance their resistivities against peroxidative degradation. Following ingestion, polyphenols also putatively act as preventative agents against a range of degenerative diseases such as atherosclerosis and selected cancers, together with systemic inflammatory conditions, all of which involve excessive ‘oxidative stress’ inputs in vivo [66].

## 4. Conclusions

In conclusion, although soybean oils generated similar levels of all α,β-unsaturated aldehydes to those of near total PUFA content-equivalent corn oil when exposed to LSSFEs, significantly higher levels of low-molecular-mass *n*-alkanals were found to be formed at both the 30 and 60 min heating time-points than those observed in all other frying oils investigated. Hence, this observation appears to arise solely from the peroxidation of linolenoylglycerols and not linoleoylglycerols in soybean oil products, since they act as substrate sources of this class of aldehydic LOPs [6,15]. Indeed, the application of MV ^1^H NMR-linked chemometrics techniques confirmed this distinction, as did a relatively simple examination of the ratios of the concentrations of low- to high-molecular-mass n-alkanals generated in these oils. One orthogonal principal component (PC3) arising from a PCA analysis, which was most strongly loaded with LMM *n*-alkanals and 4-oxo-*n*-alkanals, was postulated to contain aldehydic LOPs which exclusively arise from the thermo-oxidation of linolenoyl- and not linoleoylglycerol sources. 

Limiting the exposure to LOPs by consumption of less fried food and/or the employment of peroxidatively-resistant oils which contain high or higher MUFA contents, may serve to successfully combat the deleterious health risks posed by the ingestion or inhalation of aldehydes and further LOPs generated in soybean and other PUFA-rich cooking oils during frying practices. However, the use of alternative cooking methods such as vacuum or pressure frying, the blending of natural soybean oils with alternative, albeit MUFA-rich oils, and the use of genetically-modified high MUFA (oleoylglycerol) content soybean oil varieties, could serve as useful precautions against its generation of toxic LOPs during domestic and commercial culinary frying practices. Additionally, supplementation of this frequently used cooking oil with ‘health-friendly’ and thermally resistant polyphenolic antioxidants may also offer a valuable solution to this problem. 

## Figures and Tables

**Table 1:** Chemical shift values, multiplicities and assignments of the major TAG resonances in ^1^H NMR spectra acquired on culinary oils at an operating frequency of 600 MHz. Coupling patterns are provided in brackets following the chemical shift values provided. The codes correspond to label assignments of the resonances shown in Figure 1. Abbreviations: TMS, tetramethylsilane; 2,5-DTBHQ, 2,5-dibydroxy-*tertiary*-butyl-hydroquinone (added chain-breaking antioxidant); TCB, 1,3,5-trichlorobenzene (quantitative internal standard); *s*, singlet; *d*, doublet; *t*, triplet; *dd*, doublet-of-doublets; *dt*, doublet-of-triplets; *m*, multiplet.

Label Code	Chemical Shift (δ/ppm) and Multiplicity	Assignment
1	0.00 (*s*)	TMS Si(CH_3_)_4_
2	0.84–0.91 (*t*)	Terminal-CH_3_ group of all FAs, with the exception of unsaturated Ω-3 FAs
3	0.95–0.99 (*t*)	Terminal-CH3 group of unsaturated Ω-3 FAs
4	1.20–1.40 (*m*)	Bulk-chain acyl–(CH_2_)_n_- groups of FA chains
5	1.56–1.66 (*m*)	Acyl-OCO-CH_2_-CH_2_- groups of FA chains
6	1.95–2.11 (*m*)	-CH=CH-CH_2_- protons of FA chains
7	2.26–2.36 (*m*)	-OCO-CH_2_- group protons of FA chains
8	2.75–2.79 (*dt*)	Bis-allylic-CH=CH-CH_2_-CH=CH- functions of linoleoylglycerol FA acyl chains
8	2.79–2.82 (*dt*)	Bis-allylic-CH=CH-CH_2_-CH=CH- functions of linolenoylglycerol FA acyl chains
9	4.11–4.32 (*dd*,*dd*)	Glycerol backbone-1,3-position-CH_2_OCOR
10	5.23–5.28	Glycerol backbone-2-position-CH(OH)-
11	5.30–5.40 (*m*)	Vinylic -CH=CH- protons
12	6.59 (*s*)	2,5-DTBHQ aromatic ring protons
13	7.26/7.27(*s*/*s*)	1,3,5-TCB/residual CHCl_3_

**Table 2:** Statistical significance of differences between the oil type and LSSFE sampling time-point main effect factors, and of the oil type x sampling time-point first-order interaction effect on the FA contents of culinary oils. In addition to the main factors, the latter interaction effect was very highly statistically significant for all FAs tested, and arises from differential responses of oil compositions to increasing LSSFE heating times (Figure S1). Additionally, R^2^ and Q^2^ values are provided for the mathematical model of Equation (1); R^2^ corresponds to the linear predictive capacity of the Equation (1) model, whereas Q^2^ represents the goodness-of-fit of a ‘test’ set of data points originally removed from the model which was then generated from the remaining ‘training’ set.

	SFAs	MUFAs	PUFAs	Total UFAs
**R²**	0.971	1.000	0.999	0.971
**Q^2^**	0.935	1.000	0.999	0.935
**Oil Type *p* value**	5.21 × 10^−4^	2.00 × 10^−178^	2.57 × 10^−132^	5.22 × 10^−44^
**Heating Time *p* value**	4.61 × 10^−52^	1.19 ×10^−26^	1.19 × 10^−46^	4.62 × 10^−52^
**Oil Type x Heating Time Interaction *p* value**	8.25 × 10^−19^	1.48 ×10^−51^	4.86 × 10^−28^	8.27 × 10^−19^

**Table 3:** Chemical shift values, multiplicities and assignments of aldehyde-CHO function resonances in ^1^H NMR spectra acquired on thermally stressed culinary oils at an operating frequency of 600 MHz. Abbreviations: *d*, doublet; *t*, triplet.

Code	Chemical Shift (δ/ppm) and Multiplicity	Assignment (-CHO Function Resonances Only)
1	9.48–9.51 (*d*)	(*E*)-2-alkenals
2	9.52–9.54 (*d*)	(*E*,*E*)-2–4-alkadienals
3	9.54–9.57 (*d*)	4,5-epoxy-(*E*)-2-alkenals
4	9.57–9.60 (*d*)	Combined 4-hydroxy-(*E*)-2-alkenals and 4-hydroxyperoxy-(*E*)-2-alkenals
5	9.60–9.62 (*d*)	(*E*,*Z*)-2,4-alkadienals
6	9.74–9.77 (*t*)	*n*-alkanals
7	9.80–9.83 (*t*)	4-oxo-*n*-alkanals
8	9.85–9.87 (*t*)	LMM *n*-alkanals
9	10.05–10.08 (*d*)	(*Z*)-2-alkanals
10	10.13–10.18 (*d*)	Unassigned unsaturated aldehyde

**Table 4:** Aldehydic LOP loadings vectors for a three-component PCA model performed on all analytical data using Varimax rotation and Kaiser normalisation. Bold values indicate significant loadings vectors, and those highlighted in yellow represent the most strongly loaded PC for each aldehyde considered.

Aldehydic LOP	PC1	PC2	PC3
(*E*)-2-alkenals	**0.48**	**0.80**	0.33
(*E*,*E*)-2-4-Alkadienals	**0.79**	**0.44**	**0.42**
4-5-Epoxy-(*E*)-2-alkenals	**0.70**	**0.59**	**0.41**
4-OH-/4-OOH-(*E*)-2-Alkenals	**0.69**	**0.59**	0.39
(*E*,*Z*)-2,4-Alkadienals	**0.83**	0.35	**0.42**
*n*-Alkanals	**0.65**	**0.66**	0.36
4-Oxo-(*E*)-2-alkenals	**0.50**	**0.41**	**0.75**
(*Z*)-2-Alkenals	0.30	**0.71**	**0.62**
LMM *n*-Alkanals	**0.61**	0.36	**0.66**

## Data Availability

All study data will be provided to those who request this information from the correspondence author.

## Supplementary Materials

The following are available online at https://www.mdpi.com/article/10.3390/foods10102481/s1. Figure S1. Bar diagram plot of mean±95% confidence intervals (CIs) for culinary oil contents of (a) MUFAs, (b) PUFAs, (c) Total UFAs and (d) SFAs in six culinary oils exposed to LSSFEs for periods of 0-90 min. (95% CIs in (b) are not clearly visible in S1(a) in view of their extremely small values). (e), Corresponding diagram for the omega-3 FA contents of the two soybean oils investigated. Abbreviations: SBO1 and SBO2, soybean oils 1 and 2; CO1, corn oil 1; AVO1, avocado oil 1; IEVOO1 and SEVOO1, Italian and Spanish extra-virgin olive oils 1 respectively. Table S1. Relative MUFA, PUFA, ω-3, total unsaturated and saturated fatty acid contents (molar %) of culinary oils estimated by 1H NMR analysis at LSSFE time-points of 0, 5, 10, 20, 30, 60 and 90 min. Oil type abbreviations: as Figure S1. *(*w*/*w*) contents provided by literature sources; na, not applicable. Table S2. Mean aldehydic LOP concentrations (mmol./mol. FA) in culinary oils exposed to LSSFEs at 180oC FOR 0-90 min. Oil type abbreviations: as Figure S1. Table S3. Total levels of unsaturated and saturated aldehydes (mmol./mol. FA) in each culinary oil exposed to LSSFEs for periods of 0, 5, 10, 20, 30, 60 and 90 min. Oil class abbreviations: as Figure S1. Table S4. Least square mean (LSM) values for aldehydic LOP concentrations generated in soybean, corn, avocado and extra-virgin olive oils when exposed to LSSFE cycles for 0-90 min., and summary of all pairwise comparisons made to determine the significance of their differences between different culinary oil products (Bonferroni-corrected). (a), (E)-2-alkenals; (b), (E,E)-2-4-alkadienals; (c), 4,5-Epoxy-(E)-2-alkenals; (d), 4-OH-/4-OOH-(E)-2-alkenals; (e), (E,Z)-2,4-alkadienals; (f), n-alkanals; (g), 4-oxo-n-alkanals; (h) (Z)-2-alkenals; (i), low-molecular-mass (LMM) n-alkanals. For each aldehyde, culinary oil LSMs which are significantly different from each other have differing letter codes (A-E). The statistical significance of differences between individual culinary oil products were determined by post-hoc ANOVA tests performed by the Bonferroni method. Oil product abbreviations correspond to those in Figure S1.

## Abbreviations

AHC	Agglomerative hierarchical clustering
BHT	Butylated hydroxytoluene
CVD	Cardiovascular disease
DNA	Deoxyribonucleic Acid
2,5-DTBHQ	2,5-di*-tertiary*-Butylhydroquinone
FA	Fatty acid
HPLC	High-performance liquid chromatography
LMM	Low-molecular-mass
LOP	Lipid oxidation product
LSSFE	Laboratory-simulated shallow frying episode
MDA	Malondialdehyde
MUFA	Monounsaturated fatty acid
MV	Multivariate
NMR	Nuclear magnetic resonance
OPLS-DA	Orthogonal projections to latent structures-discriminant analysis
PC	Principal component
PCA	Principal component analysis
PLS-DA	Partial least-squares discriminant analysis
PUFA	Polyunsaturated fatty acid
SEM	Standard error of the mean
SFA	Saturated fatty acid
TBA	2-Thiobarbituric acid
TBARS	2-Thiobarbituric Acid Reactive Substances
TCA	Trichloroacetic Acid
TCB	1,3,5-Trichlorobenzene
TMS	Tetramethylsilane
α-TOH	α-Tocopherol
UFA	Unsaturated fatty acid
VIP	Variable importance parameter
