# Supplementary material for: Comparative 1H NMR-Based Chemometric Evaluations of the Time-Dependent Generation of Aldehydic Lipid Oxidation Products in Culinary Oils Exposed to Laboratory-Simulated Shallow Frying Episodes: Differential Patterns Observed for Omega-3 Fatty Acid-Containing Soybean Oils
Source: Foods. 2021 Oct 17;10(10):2481. doi: 10.3390/foods10102481 (PMC8535530; doi:10.3390/foods10102481)
Supplement: Supplementary file 1 [file foods-10-02481-s001.zip › foods-1353814-supplementary.pdf]

For submission to *Foods*

Title: Comparative 1H NMR-Based Chemometric Evaluations of the Time-Dependent Generation of Aldehydic Lipid Oxidation Products in Culinary Oils Exposed to Laboratory-Simulated Shallow Frying Episodes: Differential Patterns Observed for Omega-3 Fatty Acid-Containing Soybean Oils

Angela I. Wann[1,2], Benita C. Percival[1], Katy Woodason[1], Miles S. Gibson[1], Siâny Vincent[1] and Martin Grootveld[1*]

1 Leicester School of Pharmacy, DeMontfort University, The Gateway, Leicester LE1 9BH, UK; Angela.Allen@dmu.ac.uk (A.I.W.); benita.c.percival@dmu.ac.uk (B.C.P.); katy.woodason@dmu.ac.uk (K.W.);
miles.gibson@dmu.ac.uk (M.G.); P2449250@my365.dmu.ac.uk (S.V.)

2 School of Life Sciences, Pharmacy and Chemistry, Kingston University, River House, 53–57 High Street, Kingston upon Thames KT1 1LQ, UK

* Correspondence: mgrootveld@dmu.ac.uk

SUPPLEMENTARY MATERIALS SECTION

Figure S1

(a)

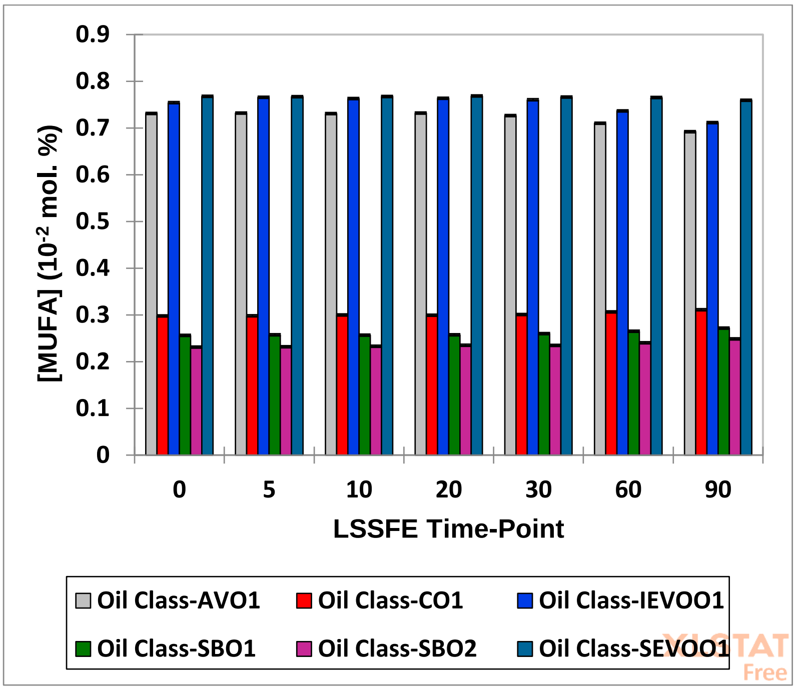

[MUFA] (10⁻² mol. %)
0.9
0.8
0.7
0.6
0.5
0.4
0.3
0.2
0.1
0
0
5
10
20
30
60
90
LSSFE Time-Point
Oil Class-AVO1
Oil Class-CO1
Oil Class-IEVOO1
Oil Class-SBO1
Oil Class-SBO2
Oil Class-SEVOO1
XLSTAT Free

(b)

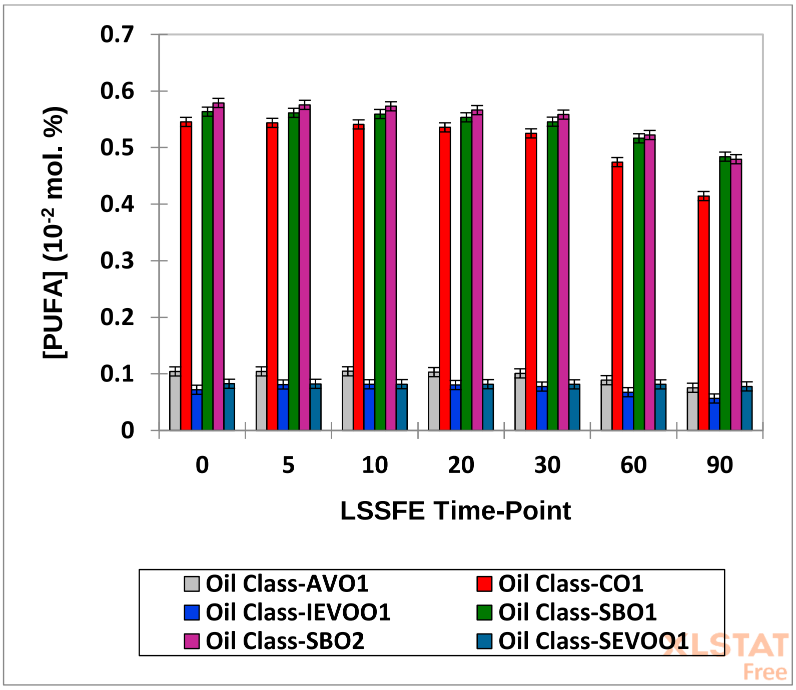

[PUFA] (10⁻² mol. %)
0.7
0.6
0.5
0.4
0.3
0.2
0.1
0
0
5
10
20
30
60
90
LSSFE Time-Point
Oil Class-AVO1
Oil Class-CO1
Oil Class-IEVOO1
Oil Class-SBO1
Oil Class-SBO2
Oil Class-SEVOO1
XLSTAT Free

(c)

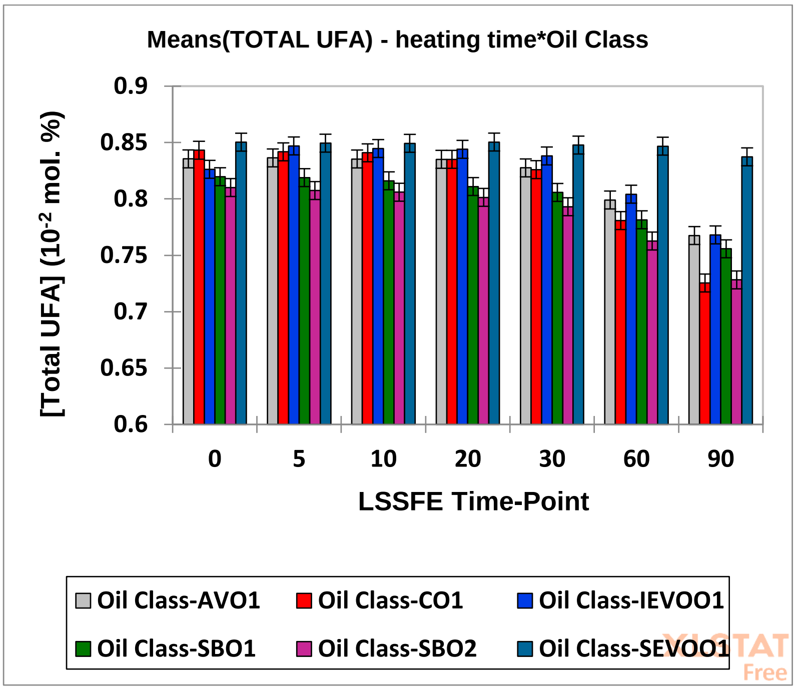

Means(TOTAL UFA) - heating time*Oil Class
[Total UFA] (10-2 mol. %)
0.9
0.85
0.8
0.75
0.7
0.65
0.6
0
5
10
20
30
60
90
LSSFE Time-Point
Oil Class-AVO1
Oil Class-CO1
Oil Class-IEVOO1
Oil Class-SBO1
Oil Class-SBO2
Oil Class-SEVOO1
XLSTAT Free

(d)

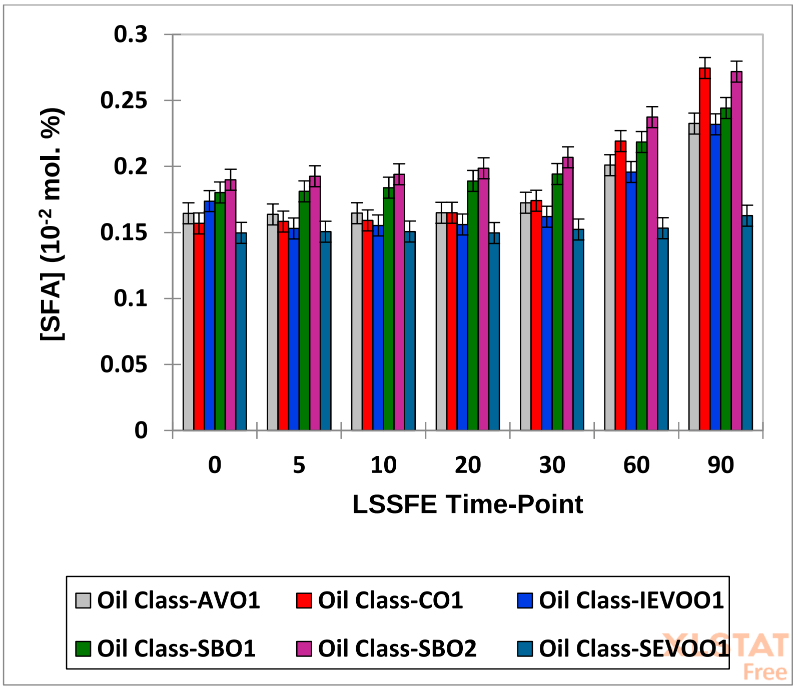

[SFA] (10-2 mol. %)
0.3
0.25
0.2
0.15
0.1
0.05
0
0
5
10
20
30
60
90
LSSFE Time-Point
Oil Class-AVO1
Oil Class-CO1
Oil Class-IEVOO1
Oil Class-SBO1
Oil Class-SBO2
Oil Class-SEVOO1
XLSTAT Free

(e)

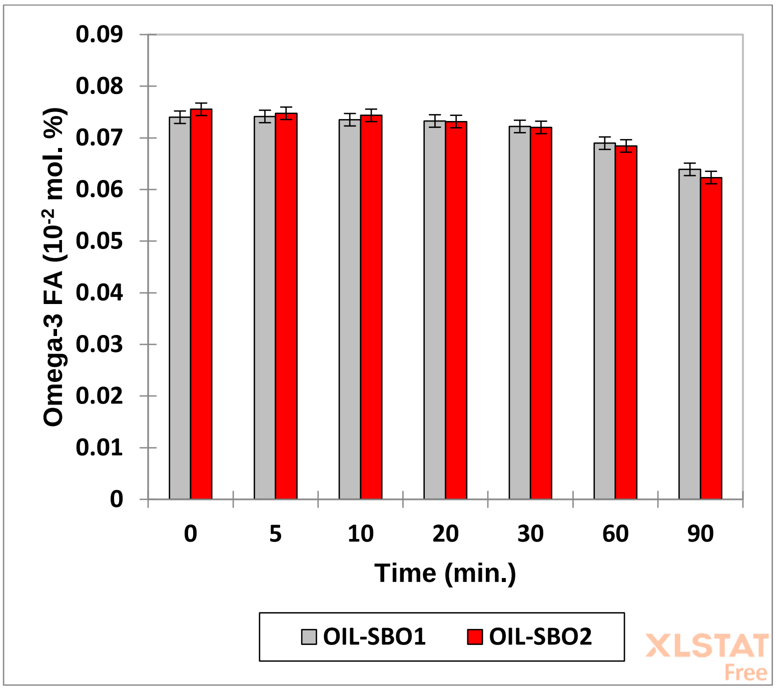

Figure S1. Bar diagram plot of mean±95% confidence intervals (CIs) for culinary oil contents of (a) MUFAs, (b) PUFAs, (c) Total UFAs and (d) SFAs in six culinary oils exposed to LSSFEs for periods of 0-90 min. (95% CIs in (b) are not clearly visible in S1(a) in view of their extremely small values). (e), Corresponding diagram for the omega-3 FA contents of the two soybean oils investigated. Abbreviations: SBO1 and SBO2, soybean oils 1 and 2; CO1, corn oil 1; AVO1, avocado oil 1; IEVOO1 and SEVOO1, Italian and Spanish extra-virgin olive oils 1 respectively.

Table S1. Relative MUFA, PUFA, ω-3, total unsaturated and saturated fatty acid contents (molar %) of culinary oils estimated by ^{1}H NMR analysis at LSSFE time-points of 0, 5, 10, 20, 30, 60 and 90 min. Oil type abbreviations: as Figure S1. *(w/w) contents provided by literature sources; na, not applicable.

Oil	Heating Time (min.)	Total MUFA Content (molar %)	Total PUFA Content (molar %)	Total UFA Content (molar %)	Total SFA Content (molar %)	ω-3 FA Content (molar %)
SEVOO1	0	76.78	8.25	85.03	14.97	0.80*
	5	76.70	8.24	84.94	15.06	na
	10	76.74	8.19	84.93	15.07	na
	20	76.86	8.18	85.04	14.96	na
	30	76.63	8.15	84.78	15.22	na
	60	76.53	8.15	84.68	15.32	na
	90	75.94	7.79	83.73	16.27	na
IEVOO1	0	75.43	7.19	82.63	17.37	0.80*
	5	76.57	8.12	84.70	15.30	na
	10	76.30	8.17	84.47	15.53	na
	20	76.36	8.03	84.40	15.60	na
	30	76.05	7.76	83.81	16.19	na
	60	75.36	7.52	82.88	17.12	na
	90	73.66	6.76	80.42	19.58	na
SBO1	0	25.62	56.35	81.97	18.03	7.40
	5	25.75	56.14	81.89	18.11	7.41
	10	25.67	55.93	81.61	18.39	7.35
	20	25.75	55.35	81.10	18.90	7.33
	30	26.00	54.58	80.57	19.43	7.22
	60	26.51	51.64	78.15	21.85	6.90
	90	27.18	48.40	75.57	24.43	6.39
SBO2	0	23.12	57.89	81.01	18.99	7.55
	5	23.19	57.55	80.74	19.26	7.48
	10	23.30	57.30	80.59	19.41	7.44
	20	23.51	56.62	80.14	19.86	7.32
	30	23.48	55.82	79.31	20.69	7.20
	60	24.03	52.41	76.44	23.56	6.84
	90	24.87	47.95	72.82	27.18	6.23
AVO1	0	73.11	10.43	83.55	16.45	0.95*
	5	73.19	10.44	83.64	16.36	na
	10	73.09	10.45	83.54	16.46	na
	20	73.20	10.31	83.51	16.49	na
	30	72.66	10.09	82.75	17.25	na
	60	71.02	8.88	79.90	20.10	na
	90	69.21	7.55	76.75	23.25	na
CO1	0	29.60	54.72	84.32	15.68	1.16*
	5	29.85	54.32	84.17	15.83	na
	10	29.85	54.24	84.09	15.91	na
	20	29.90	53.61	83.51	16.49	na
	30	30.04	52.56	82.60	17.40	na
	60	30.63	47.45	78.08	21.92	na
	90	31.17	41.37	72.54	27.46	na

Table S2. Mean aldehydic LOP concentrations (mmol./mol. FA) in culinary oils exposed to LSSFEs at 180°C FOR 0-90 min. Oil type abbreviations: as Figure S1.

**Oil**	**Heating Time (min.)**	**Concentration (mmol./mol. FA)**								
		(*E*)-2-Alkenals	(*E,E*)-2-4-Alkadienals	4,5-Epoxy-(*Z*)-2-alkenals	4-Hydroxy-/4-Hydroperoxy-(*E*)-2-alkenals	(*E,Z*)-2,4-Alkadienals	*n*-Alkanals	4-Oxo-*n*-alkanals	LMM *n*-Alkanals	(*Z*)-(2)-Alkenals
**IEVOO1**	**0**	0.026	0.060	0.001	0.004	0.004	0.045	0.000	0.000	0.000
	**5**	0.027	0.049	0.002	0.005	0.008	0.030	0.000	0.000	0.000
	**10**	0.048	0.043	0.006	0.013	0.016	0.053	0.000	0.000	0.000
	**20**	0.092	0.056	0.012	0.021	0.018	0.116	0.000	0.000	0.000
	**30**	0.256	0.112	0.033	0.023	0.029	0.255	0.000	0.000	0.000
	**60**	0.628	0.215	0.086	0.074	0.056	0.357	0.019	0.017	0.050
	**90**	1.540	0.478	0.212	0.178	0.121	0.700	0.029	0.026	0.088
**SEVOO1**	**0**	0.036	0.038	0.001	0.008	0.004	0.024	0.000	0.000	0.000
	**5**	0.025	0.041	0.000	0.014	0.008	0.025	0.000	0.000	0.000
	**10**	0.033	0.046	0.000	0.008	0.005	0.030	0.000	0.000	0.000
	**20**	0.070	0.029	0.004	0.011	0.013	0.038	0.000	0.000	0.000
	**30**	0.032	0.040	0.001	0.004	0.010	0.031	0.000	0.000	0.000
	**60**	0.041	0.033	0.003	0.011	0.013	0.049	0.000	0.000	0.000
	**90**	0.293	0.127	0.059	0.049	0.050	0.291	0.000	0.000	0.000
**SBO1**	**0**	0.051	0.045	0.012	0.014	0.021	0.094	0.000	0.000	0.000
	**5**	0.072	0.060	0.026	0.033	0.041	0.113	0.000	0.000	0.000
	**10**	0.129	0.127	0.045	0.058	0.062	0.157	0.000	0.000	0.000
	**20**	0.270	0.339	0.071	0.088	0.111	0.271	0.000	0.000	0.000
	**30**	0.556	0.709	0.159	0.170	0.205	0.493	0.000	0.000	0.000
	**60**	1.530	1.690	0.416	0.390	0.476	1.070	0.088	0.047	0.057
	**90**	2.660	2.450	0.616	0.589	0.529	1.400	0.142	0.053	0.092
**SBO2**	**0**	0.054	0.057	0.020	0.036	0.032	0.051	0.000	0.000	0.000
	**5**	0.089	0.106	0.037	0.039	0.049	0.091	0.000	0.000	0.000
	**10**	0.151	0.215	0.060	0.056	0.099	0.150	0.000	0.000	0.000
	**20**	0.402	0.564	0.127	0.120	0.218	0.342	0.000	0.000	0.000
	**30**	0.679	0.909	0.204	0.166	0.318	0.569	0.000	0.000	0.000
	**60**	1.930	2.190	0.517	0.395	0.672	1.300	0.106	0.050	0.084
	**90**	3.580	3.350	0.894	0.666	0.971	2.050	0.216	0.108	0.132
**AVO1**	**0**	0.047	0.025	0.015	0.020	0.013	0.062	0.000	0.000	0.000
	**5**	0.041	0.024	0.009	0.016	0.009	0.074	0.000	0.000	0.000
	**10**	0.072	0.037	0.018	0.018	0.012	0.099	0.000	0.000	0.000
	**20**	0.181	0.101	0.043	0.043	0.036	0.232	0.000	0.000	0.000
	**30**	0.470	0.238	0.078	0.062	0.060	0.437	0.020	0.017	0.018
	**60**	1.640	0.589	0.240	0.191	0.136	0.881	0.064	0.021	0.029
	**90**	3.210	0.795	0.367	0.311	0.146	1.260	0.102	0.028	0.058
**CO1**	**0**	0.80	0.126	0.042	0.032	0.057	0.143	0.000	0.000	0.000
	**5**	0.063	0.111	0.037	0.031	0.047	0.149	0.000	0.000	0.000

	**10**	0.091	0.117	0.039	0.030	0.050	0.167	0.000	0.000	0.000
	**20**	0.231	0.265	0.073	0.057	0.09	0.310	0.000	0.000	0.000
	**30**	0.555	0.599	0.156	0.130	0.206	0.564	0.000	0.000	0.000
	**60**	2.310	2.030	0.514	0.393	0.580	1.370	0.136	0.041	0.123
	**90**	4.500	3.050	0.887	0.676	0.780	2.120	0.211	0.058	0.209

Table S3. Total levels of unsaturated and saturated aldehydes (mmol./mol. FA) in each culinary oil exposed to LSSFEs for periods of 0, 5, 10, 20, 30, 60 and 90 min. Oil class abbreviations: as Figure S1.

Aldehyde Class	Oil Type	Time (min.)						
		0	5	10	20	30	60	90
		Aldehyde Concentration (mmol./mol. FA)						
Unsaturated	IEVOO1	0.09	0.09	0.13	0.20	0.45	1.10	2.62
	SEVOO1	0.09	0.09	0.09	0.13	0.09	0.10	0.58
	AO1	0.15	0.15	0.19	0.34	0.95	3.12	4.76
	SBO1	0.14	0.23	0.42	0.88	1.80	4.56	6.95
	SBO2	0.20	0.30	0.58	1.43	2.28	5.82	9.67
	CO1	0.34	0.29	0.33	0.72	1.65	5.95	10.10
Saturated	IEVOO1	0.04	0.03	0.05	0.12	0.26	0.42	0.75
	SEVOO1	0.02	0.03	0.03	0.04	0.03	0.05	0.29
	AO1	0.19	0.20	0.23	0.32	0.55	1.22	1.48
	SBO1	0.09	0.11	0.16	0.27	0.49	1.21	1.60
	SBO2	0.05	0.09	0.15	0.34	0.57	1.62	2.37
	CO1	0.14	0.15	0.17	0.31	0.56	1.54	2.39

Table S4. Least square mean (LSM) values for aldehydic LOP concentrations generated in soybean, corn, avocado and extra-virgin olive oils when exposed to LSSFE cycles for 0-90 min., and summary of all pairwise comparisons made to determine the significance of their differences between different culinary oil products (Bonferroni-corrected). (a), (*E*)-2-alkenals; (b), (*E,E*)-2-4-alkadienals; (c), 4,5-Epoxy-(*E*)-2-alkenals; (d), 4-OH-/4-OOH-(*E*)-2-alkenals; (e), (*E,Z*)-2,4-alkadienals; (f), *n*-alkanals; (g), 4-oxo-*n*-alkanals; (h) (*Z*)-2-alkenals; (i), low-molecular-mass (LMM) *n*-alkanals. For each aldehyde, culinary oil LSMs which are significantly different from each other have differing letter codes (A-E). The statistical significance of differences between individual culinary oil products were determined by *post-hoc* ANOVA tests performed by the Bonferroni method. Oil product abbreviations correspond to those in Figure S1.

**(a)**						
**Culinary Oil**	**LSMs ((*E*)-2-Alkenals)**	**Significantly Different Groups**				
**SEVOO**	0.08	A				
**IEVOO**	0.71		B			
**SBO1**	0.75		B	C		
**AVO1**	0.81		B	C		
**SBO2**	0.98			C	D	
**CO1**	1.12				D	
**(b)**						
**Culinary Oil**	**LSMs ((*E*,*E*)-2-4-Alkadienals)**	**Groups**				
**SEVOO1**	0.05	A				
**IEVOO1**	0.20		B			
**AVO1**	0.26		B			
**SBO1**	0.77			C		
**CO1**	0.90			C		
**SBO2**	1.06				D	
**(c)**						
**Culinary Oil**	**LSMs (4,5-Epoxy-(*E*)-2-alkenals)**	**Groups**				
**IEVVOO1**	0.01	A				
**SEVOO1**	0.08		B			
**AVO1**	0.11		B			
**SBO1**	0.19			C		
**CO1**	0.25				D	
**SBO2**	0.27				D	
**(d)**						
**Culinary Oil**	**LSMs (4-OH-/4-OOH-(*E*)-2-Alkenals)**	**Groups**				
**SEVOO1**	0.02	A				
**IEVOO1**	0.08		B			
**AVO1**	0.09		B			

**SBO1**	0.19			C		
**CO1**	0.19			C		
**SBO2**	0.21			C		
**(e)**						
**Culinary Oil**	**LSMs ((*E*,*Z*)-2,4-Alkadienals)**	**Groups**				
**SEVOO1**	0.01	A				
**IEVOO1**	0.05	A	B			
**AVO1**	0.06		B			
**SBO1**	0.21			C		
**CO1**	0.26				D	
**SBO2**	0.34					E
**(f)**						
**Culinary Oil**	**LSMs (*n*-Alkanals)**	**Groups**				
**SEVOO1**	0.07	A				
**IEVOO1**	0.32		B			
**AVO1**	0.43			C		
**SBO1**	0.51			C		
**SBO2**	0.65				D	
**CO1**	0.69				D	
**(g)**						
**Culinary Oil**	**LSMs (4-Oxo-*n*-alkanals)**	**Groups**				
**SEVOO1**	0.00	A				
**IEVOO1**	0.01	A	B			
**AVO1**	0.01	A	B	C		
**SBO1**	0.03		B	C	D	
**SBO2**	0.05			C	D	
**CO1**	0.05				D	
**(h)**						
**Culinary Oil**	**LSMs ((*Z*)-2-Alkenals)**	**Groups**				
**SEVOO1**	0.00	A				
**IEVOO1**	0.02	A	B			
**SBO1**	0.02	A	B			
**AVO1**	0.03		B	C		
**SBO2**	0.03		B	C		
**CO1**	0.05			C		
**(i)**						
**Culinary Oil**	**LSMs (LMM *n*-Alkanals)**	**Groups**				
**SEVOO1**	0.00	A				
**IEVOO1**	0.01	A	B			

**AVO1**	0.01		B	C		
**CO1**	0.01		B	C	D	
**SBO1**	0.02			C	D	
**SBO2**	0.02				D	
